# Exosome Engineering for Blocking Gut Dysbiosis and Inducing Cell Death Mechanisms in Glioblastoma Multiforme

**DOI:** 10.3390/cells15050422

**Published:** 2026-02-27

**Authors:** Ahalya Muraleedharan, Karthik Rangavajhula, Swapan K. Ray

**Affiliations:** 1 Department of Chemistry and Biochemistry, University of South Carolina, Columbia, SC 29208, USA; ahalya@email.sc.edu (A.M.); kvrangavajhula@gmail.com (K.R.); 2 Department of Pathology, Microbiology, and Immunology, University of South Carolina School of Medicine, 6439 Garners Ferry Road, Columbia, SC 29209, USA

**Keywords:** glioblastoma multiforme, exosome engineering, exosome biogenesis and cargo loading, gut dysbiosis, apoptosis, ferroptosis

## Abstract

Glioblastoma multiforme (GBM) is the most lethal primary brain tumor in adults. Emerging evidence endorses that gut dysbiosis contributes to GBM progression through the gut–brain axis (GBA), promoting inflammation and therapeutic resistance via abnormal short-chain fatty acid production and cytokine dysregulation. Exosomes, naturally occurring nanovesicles (30–150 nm), offer promising therapeutic potential due to their blood–brain barrier permeability, biocompatibility, and versatile cargo capacity. This review examines exosome engineering strategies for dual targeting: inhibiting alterations in gut microbiome and inducing regulated cell death mechanisms such as apoptosis and ferroptosis in GBM. We describe exosome engineering with detailed focus on cargo loading approaches (e.g., genetic modification, electroporation, and sonication), exosome surface functionalization with specific ligands (e.g., antibodies), and exosome biogenesis pathway manipulation. Engineered exosomes can deliver anti-inflammatory agents and gut microbiome modulators to restore GBA homeostasis while simultaneously transporting tumor-suppressive non-coding RNAs (e.g., miRNAs, siRNAs) and therapeutic agents to induce apoptosis by overcoming temozolomide resistance, and trigger ferroptosis-inducing components in GBM stem cells. Preclinical studies make obvious that this dual-targeting approach ought to enhance therapeutic efficacy by creating systemic immunity and eliminating tumor cells. However, clinical translation brings forth challenges, such as manufacturing, targeting specificity, and standardized quality control, and warrants further study.

## 1. Introduction

Glioblastoma multiforme (GBM) is the most common and malignant brain tumor in adults [1]. GBM is usually derived from astrocytoma and designated as the grade IV malignancy according to the World Health Organization (WHO) [2]. It is difficult to define the global incidence of GBM, as it can vary depending on the origin of reports. However, a recent epidemiological report denotes that the rate of occurrence of GBM among other brain tumors is 47.7% and it has an incidence of 3.21 per 100,000 population [3]. The survival of a GBM patient is about 15 months with no cure available yet [4]. According to the Statistical Report from the Central Brain Tumor Registry of the United States (CBTRUSs) on the epidemiology of primary brain and central nervous system (CNS) tumors, the occurrence of GBM among children is very low, and the reports state the likelihood of GBM diagnosis is 1.57% higher in men than women [5]. It has two subtypes: primary GBM (pGBM) and secondary GMB (sGBM). The pathogenesis, age range, molecular profiles, and other factors vary between the two subtypes [6]. pGBM develops de novo without precursor lesions, which progress into diffuse astrocytomas (WHO grade II) [7,8]. The older population is most prominently affected by pGBM along with overexpression of epidermal growth factor receptor (EGFR, a tumor promoter), mutations in PTEN/MMAC1 (phosphatase and tensin homolog/mutated in multiple advanced cancers 1, a tumor suppressor), deletions of cyclin dependent kinase inhibitor 2A (CDKN2A/p16, a tumor suppressor), and amplification of MDM2 (murine double minute 2, a critical negative regulator of the tumor suppressor p53) being the prominent characteristics of pGBM [6]. sGBM progresses from low-grade diffuse astrocytoma or anaplastic astrocytoma (WHO grade III) [7,8] Also, sGBM mostly forms in younger population while having TP53 gene (which encodes p53) mutations as a marker [6]. Another difference between pGBM and sGBM is the presence of isocitrate dehydrogenase 1 (IDH1) mutations as dominant markers in sGBM compared to being rarely present in pGBM [8].

The key feature of diffuse gliomas (astrocytomas, oligodendrogliomas, and GBMs) is the tumor cell infiltration throughout the neuropil in the gap between neuronal and glial cell bodies that contain dendrites, axons, synapses, and glial cell processes [9,10]. In gliomas, the neuropil becomes invaded by infiltrative growth of tumor cells, which is unique and distinct compared to other tumor types [11]. Hence, the presence of diffuse infiltrative growth in brain tissue is an indicator of diffuse glioma [12]. Specifically, diffuse glioma patterns are arranged into secondary structures, such as perineuronal growth, surface/subpial growth, perivascular growth, and interfascicular growth, using the structure of pre-existing tissue elements [12,13]. Apart from secondary structures, the neoplastic cells also use neuropil structures to travel long distances [9]. When comparing the aggressiveness of different gliomas, GBM appears to be the most aggressive because of its high infiltration rate. GBM also includes varying tumor patterns, such as small cell, giant cell, gliosarcoma, etc. [9]. The infiltration rate that defines GBM is like the activity of guerrilla warriors due to its randomness and aggressiveness [11]. Typically, the tumor cells act individually or in smaller clusters and attack the neuropil; however, in larger diffuse gliomas, the tumor cells take over pre-existing delivery lines of oxygen and other nutrients that increase the efficiency of their attacks [11]. The diffuse infiltration results in neuronal hyperexcitability, seizures, blood vessel dysfunction, and disruption of the metabolic state of the brain [14]. The method of invasion is still unknown, and researchers are relentlessly chasing the components involved in this process because uncovering the definite infiltration mechanism will allow the creation of a therapeutic drug targeting the cascade. There are several standard cancer therapies in use for GBM: surgery, radiation therapy, and chemotherapy [15]. A standard pharmacological oral agent for GBM is temozolomide (TMZ) [16]. However, there are novel techniques currently undergoing development, such as immune checkpoint blockade, chimeric antigen receptor T (CAR T) cell therapy, oncolytic virotherapy, and vaccine therapy for the treatment of GBM [15].

The regulation of the gut–brain axis (GBA) has been explored in neurodegenerative diseases such as Alzheimer’s disease (AD), Parkinson’s disease (PD), multiple sclerosis, epilepsy, etc. However, the implications of the GBA have been rarely studied in the realm of neuro-oncology that relates to gliomas, especially GBM [16]. According to a study targeting the global burden of cancers, infectious agents—encompassing oncogenic viruses and bacterial taxa found in the human microbiome—collectively contribute to about 20% of cancers worldwide, underscoring the etiologic relevance of the host-associated microbes in carcinogenesis [17]. There are established differences in the composition of the gut microbiome (also called gut microbiota) between glioma patients and other patients, which further strengthens the relationship between the gut microbiome and the regulation of pathogenesis of brain cancers [16]. Hence, exploring therapeutic methods to regulate the implications of the gut microbiome on GBM pathogenesis is useful because the products of gut dysbiosis, which is an imbalance in the composition of gut microbiome, directly affect brain health and diseases. One of the challenges for GBM therapy is bypassing the blood–brain barrier (BBB). A reasonable method to overcome the BBB is through the usage of exosomes, which are non-invasive, potentially natural drug-delivery nanovehicles, and derived from different cells [18]. Exosomes are identified to help us regulate gut dysbiosis apart from being implicated in GBM [18,19]. This article will focus on description to examine the potential use of exosomes for their mechanistically promising, yet untested, capacity to attenuate gut dysbiosis and promote the regulated cell death (RCD) mechanisms such as apoptosis and ferroptosis in GBM.

## 2. Limitations of Current Therapeutic Options for GBM

The GBM treatments used over the decades have various limitations because of the robustness and strength of these tumor cells. One of the standard options for cancer treatment is chemotherapy using the alkylating agent TMZ. However, this treatment faces strong chemoresistance due to the properties of the GBM stem cells (GSCs) in the tumor [20]. Among the first studies to report the presence of cancer, stem cells explored breast cancer and concluded the point of tumorigenic CD44+CD24-/low lineage to form tumors in the mice [21]. Stem cells are self-renewing cells that can differentiate into multiple cell lines with different functions based on the location of the cells [22]. According to the hierarchical cancer stem cell (CSC) model of cancer, mutations in functional progenitor cells accumulate CSCs in the tumors resulting in chemoresistance [23]. The current therapeutic options for killing GSCs and GBM cells need to be much improved to become more efficient and to have fewer side effects. Apart from strong chemoresistance, other challenges include incomplete resection, a high degree of intertumoral and intratumoral heterogeneity, BBB, and an immunosuppressive microenvironment making GBM one of the “cold tumors” that are less likely to be responsive to immunotherapy [24].

There are two different types of chemotherapy, cytotoxic chemotherapy and anti-angiogenic chemotherapy, which can be used for treatment of GBM. Cytotoxic chemotherapy consists of a post-surgical treatment with 6 weeks of concomitant TMZ (75 mg/m^2^) and radiotherapy (RT) with another dose of TMZ for six more cycles [24]. The side effects of TMZ usage include hematologic toxicity, nausea, anorexia, fatigue, and hepatoxicity [24]. The second type of chemotherapy is anti-angiogenic chemotherapy using bevacizumab. This is a humanized monoclonal antibody with anti-angiogenic properties, which binds to vascular endothelial growth factor A (VEGF-A), to prevent generation of new blood vessels. Interestingly, bevacizumab does not increase the survival rate for newly diagnosed GBM patients; hence, it is mostly used for patients undergoing recurrent GBM [24]. The side effects of bevacizumab include hypertension and leukopenia [24].

In unresectable GBM, the primary treatment method is RT [15]. External beam RT has been in use for GBM treatment for decades because it is useful for fragile populations, such as the elderly [25]. Depending on the patient, RT is combined with chemotherapy in different ways [15]. A study analyzed the efficiency of the current treatment method through a systematic review of randomized clinical trials, which resulted in the conclusion that RT in combination with TMZ could provide a better survival rate compared to the usage of RT only [26].

## 3. Gut Microbiome and Role of Gut Dysbiosis in GBM Pathogenesis

### 3.1. Gut Microbiome

**Figure 1:**
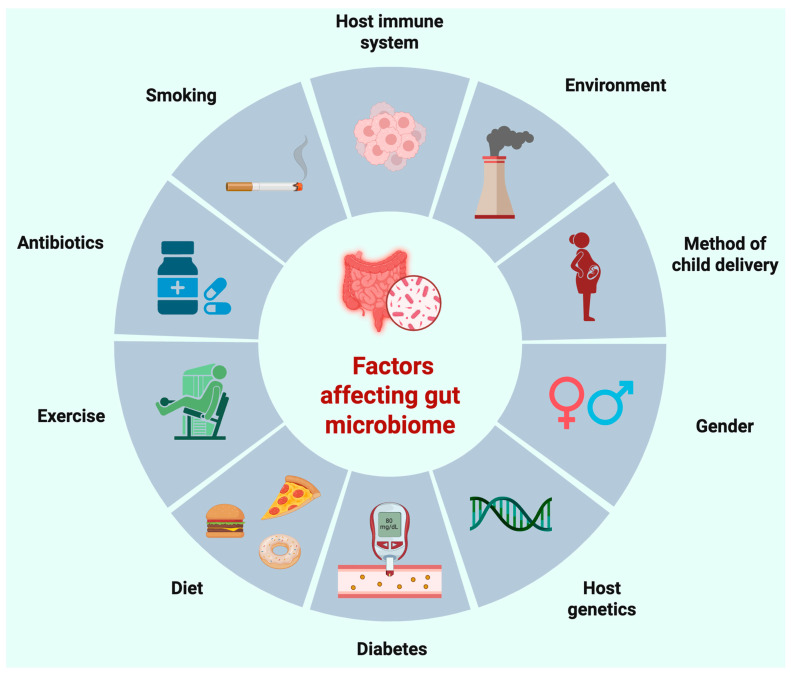
Some of the prominent factors affecting the gut microbiome in humans. This illustration details the potential factors that cause the onset of gut dysbiosis. Detrimental effects of some of the factors, such as a toxic environment, antibiotics, smoking, bad dietary habits, non-vaginal baby delivery, lack of exercise, diabetes, male gender, host defective genetics, and host weak immune system, eventually contribute to the development of gut dysbiosis. The disruption of gut microbial equilibrium can trigger systemic inflammatory responses and increase susceptibility to various metabolic and gastrointestinal disorders. Understanding the multifaceted drivers of gut dysbiosis increasingly appears to be highly essential for developing targeted therapeutic interventions aimed at restoring intestinal homeostasis and preventing consequences of gut dysbiosis in pathogenesis of diseases in other organs. This figure was created using BioRender.

The gut microbiome consists of microorganisms flourishing in the human gastrointestinal tract. The gut flora present in everyone is unique, resulting from varying bacterial compositions, affecting diseases, immunity, etc. This community of microorganisms, the microbiota in the gut, leans heavily toward prokaryotes with less emphasis on fungi, parasites, and archaea [27]. There are many factors that need to be normal for a normal gut microbiome in humans (Figure 1).

Alterations in one or more factors have the potential to cause imbalance in the gut microbiome, leading to gut dysbiosis that affects health and the course of diseases. The population and diversity of the flora depend on the geographical location of the gut microbiome being examined. The bacteria in the microbiome are also classified through phylogenetic nomenclature into taxonomic ranks stratified based on genetic similarity [27]. The human gut contains approximately 1000 microbial species, with 2,000,000 bacteria compared to about 20,000 human genes [28]. Among the bacterial phyla present in the gut, *Firmicutes* (new name *Bacillota*) and *Bacteroides* (new name *Bacteroidata*) account for 3/4th of the population [29]. Other than microorganisms, the gut microbiome also includes structural elements (e.g., nucleic acids, proteins, lipids, and polysaccharides), metabolites (e.g., signaling molecules, toxins, and organic and inorganic molecules), and other molecules produced by the gut microbiota [30]. Additionally, there are conflicts about whether viruses, phages, and extracellular DNA should be included in the umbrella term of “microbiome” [30]. The diversity of the gut microbiome is influenced by cultural and regional lifestyle differences along with medication usage, stress levels, physical activity, and sleep–wake cycles [31]. The diverse gut flora is being explored in terms of discovering origin of a multitude of diseases because of its immunological, metabolic, and endocrine cascades implicated in their pathogenesis.

### 3.2. Gut–Brain Axis

**Figure 2:**
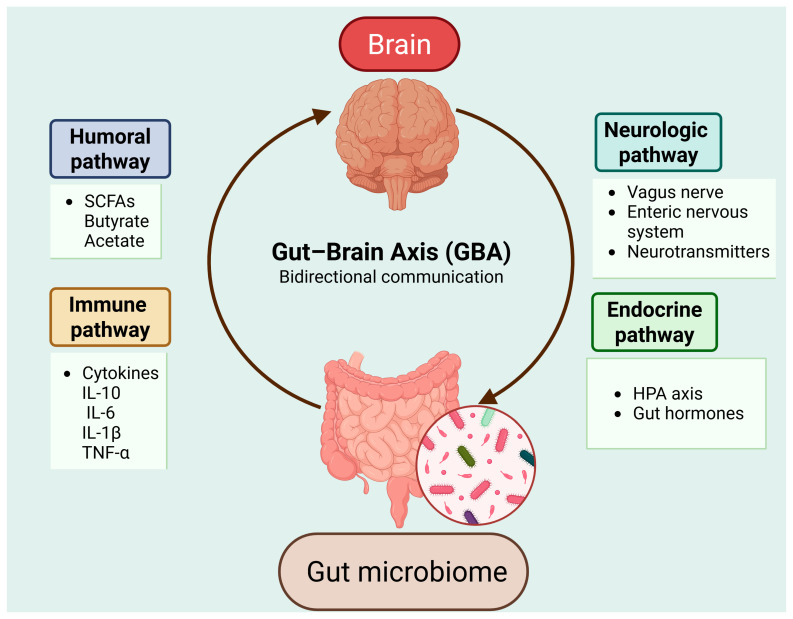
The relationship between the brain and the gut microbiome via gut–brain axis (GBA). The GBA connecting the gut microbiome and the central nervous system (CNS) plays very important roles in humoral, immune, neurologic, and endocrine pathways. The humoral pathway regulates short-chain fatty acids (SCFAs). The immune pathway regulates cytokines. The neurologic pathway involves the vagus nerve, enteric nervous system (ENS), and neurotransmitters. The endocrine pathway modules peptide production. These bidirectional communication channels allow the gut microbiota to influence cognitive functions and emotional behavior through the systemic circulation. Consequently, any imbalance in these signaling pathways may contribute to the pathogenesis of diverse neurological and psychiatric conditions. This figure was created using BioRender.

The gut microbiota is implicated in the bidirectional communication network, colloquially called the gut–brain axis (GBA) among the central nervous system (CNS), brain and spinal cord, autonomic nervous system (ANS), enteric nervous system, and the hypothalamic–pituitary–adrenal (HPA) axis [32] (Figure 2).

This network can be categorized into the neurologic pathway, the endocrine pathway, the humoral/metabolic pathway, and the immune pathway [33]. The neurologic pathway consists of the vagus nerve, the ENS, and neurotransmitters, including serotonin, dopamine, and gamma-aminobutyric acid (GABA) [33,34]. The endocrine pathway regulates the production of peptides involved in processes such as the sleep–wake cycle, blood pressure, nociception (perception of pain), etc. [33]. The immune pathway modulates the production of cytokines such as interleukin (IL)-10 and IL-4, alongside other cellular mediators. The humoral/metabolic pathway is a key pathway because it involves the production of short-chain fatty acids (SCFAs) consisting of two to six carbon atoms, three most common of SCFAs are acetate, propionate and butyrate found in human gut [33].

The bacterial fermentation of dietary carbohydrates in the gut produces SCFAs acetate, propionate, and butyrate with a 3:1:1 ratio. Most gut anaerobes produce acetate, while butyrate and propionate are produced through a glycolytic pathway, and succinate or propanediol pathway, respectively [35,36,37,38,39,40]. The general functions of SCFAs include strengthening intestinal barrier integrity, mucus production, and tolerogenic response for inflammation [41]. The SCFAs also directly affect the BBB, with studies showcasing that treating mouse with SCFAs producing bacterial strains improves the BBB and the microglia production [42,43]. SCFAs are also involved in the endocrine pathway through regulating the synthesis of gut-derived serotonin, which directly affects the GBA [44]. The components of the microbiome are heavily implicated in the pathway between the gut and the brain; hence, any imbalance and dysfunction of the gut microbiome can result in adverse effects in the brain. The ability of SCFAs to regulate neuroinflammation and the BBB can potentially have protective effects against brain diseases. Apart from regulating the BBB, SCFAs are also important in producing neural progenitor cells (NPCs) that differentiate into neuronal and glial cell types [45,46]. There are a couple of mechanisms utilized by SCFAs to increase the production of NPCs. The first mechanism uses specific free fatty acid reporters (FFAR2 and FFAR3), and the second mechanism regulates the physiological pH [47]. The SCFAs also regulate the production of cytokines, which play key roles in inflammation pathways [48]. These metabolites also modulate macrophages, neutrophils, and dendritic cells. Studies exploring the functions of microbe-derived proteins conclude that *Lactobacillus rhamnosus* GG (LGG)-derived proteins prevent cytokine-induced intestinal apoptosis [49]. LGG can inhibit the pro-inflammatory nuclear factor-kappa B (NF-κB) pathway to decrease the overproduction of inflammatory cytokines.

### 3.3. GBA and Gut Dysbiosis

An imbalance in the ratio of anti-inflammatory cytokines and pro-inflammatory cytokines due to gut dysbiosis has the potential to cause conditions such as inflammatory bowel disease (IBD), diabetes, obesity, and diverging CNS disorders [50]. Any imbalance in the gut microbiota results in gut dysbiosis, potentially leading to harmful cascades causing various diseases and disorders (Table 1).

#### 3.3.1. Gut Dysbiosis and Pathogenesis in the GBA

Gut dysbiosis is categorized into three distinct types: Type 1 involves the loss of beneficial bacteria, Type 2 is characterized by the overgrowth of pathogenic species, and Type 3 refers to a decline in overall microbial diversity [50]. This microbial imbalance is influenced by a complex interplay of genetics, clinical health, lifestyle, hygiene, and medication [62]. Diet remains a primary regulator of this equilibrium; intervention studies indicate that plant-based nutrition increases fecal SCFAs and microbial variety. Conversely, the Western diet triggers gut dysbiosis, compromises the intestinal epithelial barrier, and fuels systemic inflammation [63]. Furthermore, adequate vitamin D intake has been shown to fortify the epithelial lining, mitigating dysbiotic symptoms [63]. Environmental factors also dictate microbial composition, a process beginning at birth where delivery methods significantly shape long-term floral diversity [64]. Postnatal exposure to pollutants can further induce gut dysfunction, subsequently impairing the GBA and contributing to mental health challenges [65].

#### 3.3.2. Mechanisms of Neuroinflammation and Cognitive Decline

Metabolic imbalances within the gastrointestinal tract can lead to leaky gut, a phenomenon where pathogenic bacteria penetrate the epithelial lining to enter the bloodstream. This translocation of pathogenic bacteria triggers immunological cascades and chronic inflammation [66]. Due to the bidirectional nature of the GBA, gut dysbiosis is central to the pathogenesis of various CNS disorders. While specific pathways are still being elucidated, it is established that dysbiosis promotes bacterial amyloid formation and lipopolysaccharide (LPS) production. These factors facilitate neuroinflammation, brain atrophy, and cognitive decline [67]. Research indicates that gut dysbiosis elevates oxidative stress and insulin resistance, which are key hallmarks of AD. The gut microbiota also regulates the status of oxidative stress in the CNS via metabolites [63]; for instance, diminished levels of the SCFA butyrate can lead to mitochondrial dysfunction and increased oxidative damage [68]. In 5xFAD animal models, gut dysbiosis correlated with the CCAAT/enhancer-binding protein beta/asparagine endopeptidase pathway, which modulates AD pathogenesis through amyloid-beta and Tau proteins [69].

#### 3.3.3. Dietary Interventions and Protein Aggregation

The impact of dietary modifications—such as the Mediterranean diet, alternate-day fasting (ADF), and the ketogenic diet—on cognitive impairment via GBA modulation has been extensively studied [70]. Specifically, ADF has been shown to improve cognitive performance and anti-inflammatory profiles. In PD mouse models, these neurological benefits were directly linked to diet-induced changes in the gut microbiota [71]. Mechanistically, Gram-negative bacteria produce LPS, which stimulates cytokine release and increases intestinal permeability. These cytokines cross the BBB, activating reactive microglia and astrocytes to trigger neuroinflammatory pathways [48,67,72]. In AD, the deposition of amyloid-beta (Aβ) plaques is a defining feature. Evidence suggests that Aβ inserted into the gastric walls of mice can migrate to the brain via the vagus nerve, highlighting direct role of the gut in neurodegeneration [73]. Common gut bacteria such as *Escherichia coli* and *Staphylococcus aureus* can produce Aβ proteins. These proteins not only migrate to the brain but also activate microglia through the Toll-like receptor 2 (TLR2) pathway, exacerbating the inflammatory environment of the brain [74]. Such findings underscore the critical importance of modulating the gut microbiome to alleviate the neuropathogenesis observed in diseases ranging from AD to GBM.

### 3.4. GBA in GBM

The GBA is increasingly implicated in the CNS pathologies, particularly neurodegenerative diseases and approximately 15–20% of all cancers [17]. Recently, research has pivoted toward exploring specific role of the GBA in GBM, revealing that the microbiota significantly influences brain tumor development and progression. Gut dysbiosis serves as a direct driver of neuroinflammation [75], tumor initiation [76], and metabolic changes that facilitate tumor growth [77]. Furthermore, the microbial environment appears to modulate therapeutic responses in GBM patients [78,79], suggesting that the GBA represents a promising target for improving clinical outcomes. However, the potential for using exosomes to create a meaningful therapeutic impact on GBM via these pathways requires further experimental validation.

We are describing some GBM experimental models and microbial dynamics. Both murine and human GBM models have become essential for elucidating the complex dynamics of the GBA (Table 2), with gut dysbiosis serving as a central factor in these investigations [16]. For instance, a study comparing human and mouse GL261 GBM cells implanted in mice revealed a significant shift in microbial composition, specifically an increased *Firmicutes/Bacteroides* (F/B) ratio and a higher prevalence of the *Verrucomicrobia phylum* and *Akkermansia* genus [80]. In contrast, treatment with TMZ was found to decrease the F/B ratio, increase the *Muribaculaceae* family, and reduce the *Ruminococcaceae* family [80]. While some smaller-scale studies have reported a general decline in overall microbial diversity rather than the proliferation of specific populations [81], these findings highlight the need for larger-scale experimental and control groups to reach more definitive conclusions regarding the microbial signatures of GBM.

In a study analyzing the fecal metabolite production in GBM patients, the SCFAs (acetate, propionate, and butyrate) levels were severely decreased along with some neurotransmitters such as norepinephrine and 5-hydroxyindoleactic acid (5-HIAA) [82]. The decreased production of SCFAs also affects T cell immunity because these are crucial for regulating both effector and regulatory T cells through epigenetic and metabolic reprogramming [83]. To further strengthen the implications and association of GBA and GBM, another study concludes that antibiotics are increasing tumor growth, and antibiotics are one of the factors that cause metabolic imbalance in the gut flora [84]. In a Mendelian randomization study, other investigators used genome-wide association studies with gut microbiota and GBM. Their studies showcased an increased presence of family *Peptostreptococcaceae* and genus *Eubacterium brachy* association with GBM [85].

There are redundant patterns present across human and mouse models when the microbial taxa in GBM are compared across studies in a recent systematic review [86]. The gut microbiome consistently showed reduced *Firmicutes:Bacteroidetes* ratio, elevated *Akkermansia* and *Bacteroides*, decreased SCFAs and neurotransmitters, and intratumoral bacterial presence (primarily *Proteobacteria* and *Fusobacteriota*), all associated with neuroinflammation, immune dysregulation, and tumor progression. In contrast, SCFA-producing microbial taxa (e.g., *Faecalibacterium*, *Ruminococcaceae*) and probiotic genera (e.g., *Lactobacillus, Bifidobacterium*) showcased protective effects via restored gut barrier integrity, suppressed PI3K/Akt survival signaling, and enhanced anti-tumor immunity. Similarly, another systematic review analyzed the taxa present in AD [87]. The results are similar in terms of the decrease in *Firmicutes*; however, there was an increase in *Bacteroidetes* and *Proteobacteria* [87]. The composition of the microbiota also varies among clinical PD subtypes: lower *Firmicutes* and *Firmicutes:Bacteroidetes* ratio for tremors, and decreased *Bifidobacteria* for tremors and postural instability and gait disability (PIGD) phenotype [88]. The range of different compositions among pathologies and phenotypes suggests that, depending on the brain tumor type, the microbial taxa would differ.

The gut microbiome also influences responses to brain tumor therapies including chemotherapy, RT, and immunotherapy [89,90,91,92,93,94]. One study mentioned that modulating the gut microbiota could permit an increase in efficacy and decrease in toxicity of chemotherapeutic agents [89]. The strategies surround the planned use of dietary interventions with antibiotics, probiotics, prebiotics, synbiotics, FMI, engineered bacteria, and bacteriophages [89]. For example, probiotics release SCFAs, which improve the response of chemotherapeutic agents. Similarly, it is of immense importance to analyze manners in which the gut microbiota can be harnessed to target the neuropathology present in GBM.

Investigators have established groundbreaking foundation for the potential for gut microbiota to alleviate the immunotherapy resistance in GBM [95]. Using a genetically altered murine model, the researchers tracked the changes in the gut flora throughout tumor progression, while also using tryptophan as a dietary intervention to restore gut dysbiosis. Apart from restoring the microbial alterations, tryptophan also increases CD8+ T cell circulation in the bone marrow and the efficacy of immunotherapy. *Duncaniella dubossi* has been identified as the key bacterial species because it recreates immune responses of tryptophan. The reversal of T cell circulation proves that altering immunotherapies can result in a potential revamp for such an immunologically cold tumor as GBM [95]. Further studies need to explore other potential metabolites via the gut microbiota to target some of the unique features in the brain.

GBA and the importance of the gut flora need to be further studied in GBM with a wider range of participants and animal models, because there is a wide gap in novel studies. With the possibility of different brain tumor types presenting with varying microbial flora, the studies would also need to further break down the differences in the flora composition in different brain tumor types.

## 4. Exosomes and Their Roles in Diverse Physiological Processes

Exosomes are lipid-based membranous pockets that are part of broad category known as extracellular vesicles (EVs). Despite only recently coming into focus for their inherent potential for therapeutics, exosomes (and EVs as a whole) have been known as almost ubiquitous participants in a wide range of physiological processes necessary for human growth, development, and homeostasis.

### 4.1. Heterogeneity of EVs

**Figure 3:**
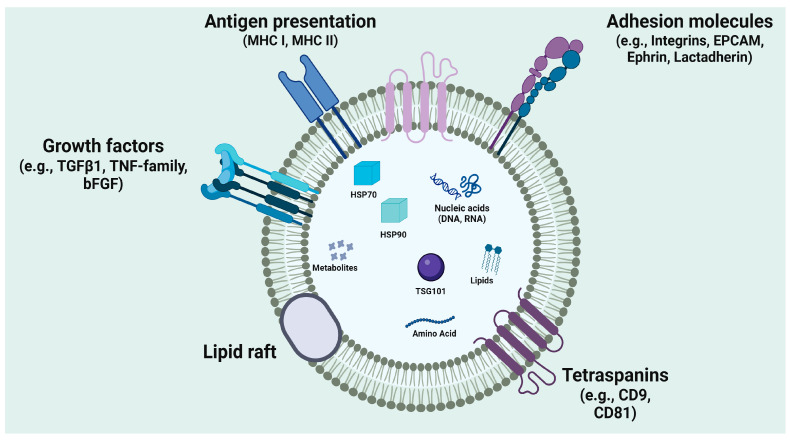
The general structure of an exosome. Various components in exosome structure play important roles in various biological functions. The structure and components of an exosome depend on the cell type. Exosomes contain membrane proteins known as tetraspanins, which include CD9, CD81, and CD63. Exosomes also have a dense intraluminal core of scaffolding and aggregating protein bound with chaperones, such as heat shock proteins 70 kDa (HSP70) and HSP90. Common cargoes present in exosomes include RNA and DNA along with lipids. These diverse molecular cargoes facilitate intercellular communication by transporting genetic and proteomic signals to specific recipient cells. Furthermore, the distinct lipid bilayer and surface proteins protect these internal components from enzymatic degradation during systemic circulation. This figure was created using BioRender.

All cells in the human body are capable of producing EVs, a process that is thought to be conserved to some degree across all domains of life [96]. While in prokaryotes, EV formation typically involves a simple budding of the cell membranes for horizontal gene transfer between unicellular organisms, EVs in humans contribute to more complex, multi-level mechanisms across the body [97]. Like their prokaryotic counterparts, human EVs have functions that center around coordination of cellular communication. EVs have the capacity to transport a range of molecules across the cellular microenvironment, including nucleic acids with gene silencing capacities, immunomodulatory cytokine molecules, and growth-stimulating protein products [98]. The similarity of EV structure to that of the cell membrane enables it to enter the cell relatively unhindered through the same processes that mediate endocytosis (Figure 3).

EVs are known to induce effects by binding to membrane receptors [99]. Signaling facilitated by EVs has a strong influence in the direction of progenitor differentiation, cell survival and proliferation, and management of cell waste [99]. Additionally, EVs play an especially crucial role in engaging or suppressing action of key immune cells in a variety of mechanisms. Due to the versatile presence of EVs among such a broad range of cellular and system processes, EVs themselves can often be used as lynchpins in the progression of diseases that target the systems [100]. In much the same fashion, the functional capacity of EVs can also be harnessed for our own purposes. Beyond their role in scientific and diagnostic applications, the use of these structures as delivery vehicles has become a research area of increasingly rich potential. Given their cellular origin, EVs have an inherent level of biocompatibility and stability through intercellular transport that often exceeds that of synthetic nanoparticle delivery systems in physiological contexts [101]. For this reason, the role of different EV types has become a highly explored topic in recent years in either induction or treatment of diseases, including GBM.

Subgroups of EVs with varying characteristics and therapeutic potential have been defined. By size, two broad categorizations have been suggested by the minimal information for studies of EVs [102]. EVs with sizes up to 200 nm have been termed as small EVs (sEVs), while the rest of EVs fall into the category of large EVs (lEVs), with sizes reaching as large as 4000 nm. Certain groups have the capacity to form vesicles of varying sizes as they have the capacity to form outwards from the outer cell membrane as ectosomes. Notably, these groups include microvesicles (100–1000 nm in diameter) that bud directly from the plasma membrane, and apoptotic bodies (50–5000 nm in diameter), which are formed from the fragmented blebbing of dying cells. Though plentiful, capable of carrying large molecules due to their sizes, and serviceable as useful medium of communications in certain contexts, both apoptotic bodies and microvesicles face certain challenges that have questioned their value from a therapeutic standpoint. The formation of both subtypes, being a budding process from external membrane, are not governed by regulatory systems [103]. Apoptotic bodies, as byproducts of apoptotic disintegration of cells, typically contain an unspecified collection of cellular components that only undergo cargo selection with specificity limited to the organelle level. Furthermore, apoptotic bodies often contain inflammatory markers, such as danger-associated molecular patterns (DAMPs), and therefore are naturally designed to use DAMPs to initiate immune clearance to protect the organism, limiting their use as therapeutics [104]. While microvesicles offer a more controlled approach to production, the contents and presented surface molecules are dependent on the specific membrane and cytoplasm content at the site of formation in the cell of origin [105]. While the options to engineer surface markers and localize cargo molecules to membrane anchor molecules lend themselves to enhancing the functional capacity of the microvesicle, these attempts can struggle to translate to therapeutic viability due to the extensive lipid reorganization process that is characteristic of budding [106]. This dynamic reorganization can drastically shift the profile of the membrane intended to form the vesicle surface, thereby disrupting the stability of molecules of interest within the membrane and their supporting lipid raft structures [107]. As a result, attempts to generate specifically designed populations for therapeutic purposes may only produce a heterogenous yield. Furthermore, this lipid rearrangement process also destabilizes the membrane structure of the microvesicle itself by shifting lipid markers like phosphatidylserine (PS) and phosphatidylethanolamine (PE) to the outer leaflet of the membrane; thereby, both PS and PE can increase the risk of structural degradation and immune clearance. From a therapeutic standpoint, exosomes are obviously more valuable than other EVs. Therefore, a deeper understanding of the exosome properties, biogenesis, and cargo loading is warranted before diving into exosome engineering for using them in alleviating gut dysbiosis and facilitating treatment for induction of RCD in GBM.

### 4.2. Exosomes and Their Properties

Of the sEV categorization, a certain subgroup known as exosomes demonstrates a stronger capacity to potentially circumvent the challenges for therapeutic use, as mentioned above. Secreted by most cell types into bodily fluids, exosomes are nanoscale vesicles that deliver bioactive genetic and proteomic material to target cells. This lipid bilayer encapsulation protects the cargo from enzymatic damage, ensuring stable systemic circulation and effective phenotypic modification. All the advantageous properties of exosomes over other types of EVs mentioned above are a product of its biogenesis pathway. Primarily, biogenesis of exosomes containing intracellular molecules is a much more intentional process than it is for its microvesicle counterparts [108].

#### 4.2.1. Initiation of Exosome Biogenesis

There is Endosomal Sorting Complex Required for Transport (ESCRT) complex that consists of several proteins, which can recognize key signaling molecules that designate cargo for exosomal integration [109]. Namely, the ESCRT-0 subcomplex of ESCRT can recognize mono-ubiquitinated proteins and, with the help of phosphatidylinositol 3-phosphate (PI3P), localize them to the membranes of the late endosome, a specialized transport organelle [110]. Here, the ubiquitinated protein is clustered to prepare for sorting into small internal structures within the late endosome known as intraluminal vesicles (ILVs). As a part of this process, other members of the ESCRT group are recruited to the late endosome to structurally stabilize the intended cargo (ESCRT-I and -II) and initiate the membrane remodeling process necessary to begin budding the late endosome into ILVs. The action of the final component of the ESCRT complex, the Vacuole Protein Sorting 4 (VPS4) ATPase, helps remove the ubiquitin tag from the cargo, recycle the ESCRT machinery, and provide energy to complete ILV scission [111]. ILVs are contained in the lumen of the late endosome, now collectively known as a multivesicular body (MVB).

#### 4.2.2. Exosome Cargo Selection

The in-built capacity of exosomes to tag molecules for packaging enables cargo selection with greater levels of reliability than other EVs. RNA-based cargo can utilize the service of certain RNA-binding proteins (RBPs) to direct them to exosomal loading sites. Exosomal non-coding RNAs (ncRNAs) such as microRNAs (miRNAs, about 22 nucleotides in length), long ncRNA (lncRNA, more than 200 nucleotides in length), and circular RNAs (circRNAs, circular molecules with a covalently closed loop structure, lacking a poly A tail) are highly stable to act as messengers in cell–cell communications. Almost all RNAs in cells exist as ribonucleoprotein complexes. RBPs are critical for promotion of ncRNA transmission in parent cells and can serve as the intracellular inducers of ncRNA loading in exosomes in the recipient cells. Also, short nucleotide sequences on RNA can guide its passage to diverse subcellular compartments, including exosomes. High enrichment of some ncRNAs in exosomes secreted by specific cells indicates that encapsulation of exosomal ncRNA is required for initiating exosomal ncRNA signaling. RBPs mediate sorting of ncRNAs depending on their characteristic motifs. The post-translational attachment of small ubiquitin-like modifier (SUMO) to proteins is essential in mediating ESCRT dependent sorting into exosomes. The miRNAs that are phosphorylated, SUMOylated, or enriched in exosome motifs (EXOmotifs) and RBP-specific motifs have the higher likelihood of selective incorporation into exosomes [112]. The ubiquitination status of proteins helps dictate their recognition by ESCRT mechanisms. Beyond ubiquitin tag detection, molecules can be directed towards sorting by ESCRT mechanisms by several other pathways.

Several cytokine receptors have demonstrated the capacity to interact directly with certain subunits of the ESCRT complex (typically the Hrs subunit of ESCRT-0) to initiate encapsulation within an ILV [105]. Apoptosis-Linked Gene 2-Interacting Protein X (ALIX) is a key multifunctional protein associated with several ESCRT processes and has been observed to interact with specific molecule domains to trigger packaging into exosomes without ubiquitination [113]. This interaction with ALIX has commonly included the YPX_3_L (tyrosine, proline, any three amino acids, and leucine) motif or others with sequence similarity to YPX_3_L (such as the Hepatitis A structural protein). Scaffolding proteins such as syntenin can help bridge interactions between ALIX and cargo molecules where direct interaction may not be possible [114]. Cargo tethered to the integral cell surface proteoglycan molecules known as syndecans (SDCs) are prime targets for association with ALIX via syntenin binding. The syndecan-syntenin-ALIX pathway is a key axis of exosome biogenesis that helps selectively integrate several receptor and ligand molecules [114]. In the human MCF7 breast cancer cell line, it has been observed that this pathway is subject to the influence of several key regulatory mechanisms that are notably involved in cancer development, including the activity of the ADP-ribosylation factor 6 (ARF6)/programmed cell death ligand 2 (PDL2) [115]. These broad pathways that govern cellular processes are noted to induce biogenesis of more distinct and specialized exosome subtypes as an almost direct extension of the pathway effect, at times even based on specific cellular contexts. The capacity to regulate initiation of exosome biogenesis and the properties of the produced exosomes through established regulatory systems demonstrates the potential of exosomes to generate a more homogenous population of targeted therapeutics for specific diseases such as GBM. Even beyond the canonical ESCRT-mediated biogenesis, the significant role of tetraspanins (a family of membrane proteins) in guiding exosomal loading and ceramide lipids in initiating formation of ILVs allow for predictable intervention points for dictation of certain biogenesis processes [116,117].

#### 4.2.3. Exosome Journey

The process of fate determination of the MVB, whether for exosomal release or lysosomal degradation, heavily involves Rab GTPases and depends on the properties of the specific exosomes [118]. Factors such as the presence of certain complexes on the MVB surface, modifications of MVB membrane proteins, the level of cargo ubiquitination, and the lipid profile of the membrane itself can determine the action of Rab GTPases in directing an MVB to either outcome. Furthermore, increased activity and expression of certain Rab GTPases and ESCRT subunits helps bias MVBs towards exosome production [119]. In particular, the activity of ESCRT-III subcomplex and the interplay of its accessory proteins strongly influence the formation of ILVs and determination of MVB fate [120]. Subsequent release of the ILVs within the MVB as exosomes occurs as actin filaments and microtubules facilitate shuttling of the MVB to the cell membrane. Vesicular docking of the MVB to the cell membrane is mediated by the soluble N-ethylmaleimide-sensitive factor attachment protein receptors (SNAREs) on both membrane surfaces [121]. The Rab GTPases continue to guide this process as well, by recruiting motor and tether proteins that are crucial to fusing the MVB with the cell membrane for successful exosome release.

Free exosomes can then travel through the body to reach their target cells. Exosomes possess certain properties that enable them to be successful cargo carriers out of the cell. The compact and stable membrane structure allows them to more easily avoid immune clearance, infiltrate through biological barriers, navigate dense cellular microenvironments, and enter blood vessels via endothelial transcytosis for biodistribution [122]. The membrane itself is highly ordered and enriched in certain lipid subtypes and macrostructures that enhance the structural integrity in the face of physical stress and enzymatic degradation. Embedded within the exosome membrane includes selectively recruited membrane proteins that confer stabilizing effects. Tetraspanins, in particular, have been found at higher levels on exosome surfaces [123]. These proteins are key in targeting the exosome towards specific cells—a notable advantage of exosome-based therapeutic delivery. Furthermore, tetraspanins form the foundation for tetraspanin-enriched microdomains (TEMs) that strengthen the association of membrane components [124]. Heat shock proteins (HSPs) are another common exosome membrane component that plays a more protective role in shielding from membrane damage during exposure to oxidative stress, varying pH, and temperature levels [125]. The membrane components of exosomes are also able to associate with serum proteins like albumin and lipoproteins to create a protective corona around the vesicle, conferring an additional level of protection from degradation, aggregation, and immune clearance [126]. The exosome structure also incorporates the natural surface markers and cytoskeletal components from the late endosome of the host cell from which they arise, typically conferring greater structural integrity and minimal immune activation risk [127]. These unique characteristics of exosomes define their capacity to serve as effective therapeutic delivery mechanisms.

Once reaching a target cell, exosomes typically follow three pathways of effect. First, exosome is capable of direct interaction with molecules on the external surface of the target cell to trigger a cascade of responses within the cell. Ligands embedded on the exosome membrane functioning in this capacity typically participate in survival or immune-related responses [128]. Interactions with immune cells also represent the potential for reversible or transient surface interactions that allow the exosomes to detach and continue signaling functioning following inducing the desired effect on a target cell. The second course of action involves fusion with the plasma membrane to directly release the cargo to fulfill its purpose within the target cell. The same SNARE, Rab proteins, and lipid domains that facilitate external release of exosomes also help mediate intake via membrane fusion [129]. While this seems to be the most direct way for cargo to enter the cell, it appears to be relatively uncommon for exosome uptake. Tumor cells, in particular, seem to be one of the few cell subtypes capable of effectively using this pathway, which is theorized to be associated with higher conformational rigidity of exosome membranes in response to lower pH of tumor environments. The more common pathway for exosome internalization among other cells is clathrin-mediated endocytosis. This process involves the construction of vesicles reinforced by a clathrin scaffold that captures and internalizes the exosomes before fusing with the greater endosome system in the target cell [130]. Exosomes in this pathway face the challenge of escaping the endosomal system to avoid lysosomal degradation or externalization. Approaches for the endosomal escape include a pH dependent fusion with endosomes and subsequent membrane permeabilization [131]. Alternative processes of internalization—which include caveolin-dependent endocytosis, phagocytosis, and micropinocytosis—seem to be less common and dependent on the cell-specific expression profiles of certain signaling factors [132].

### 4.3. Cargo of Exosome

Despite their relatively smaller size among other EVs, different exosomes have been identified to collectively carry several thousand distinct proteins and nucleic acids, as well as hundreds of lipid cargoes. The capacity to support a broad range of cargoes—especially more delicate ones such as nucleic acids—in organism-wide transport processes makes exosomes one of the most versatile transport mechanisms for delivering therapeutic to target cells like GBM cells. We present a broad look at the biological utilities of exosomes and the cargo they can carry (Table 3).

#### 4.3.1. Transportation of Proteins

Protein transport can happen by integration within the exosome membrane or the exosome cytoplasm. Membrane proteins can not only be useful in directing and protecting the exosome but can also interact with molecules on or within the target cells to induce desired effects. This is observed with the presentation of major histocompatibility complex (MHC) molecules or programmed death–ligand 1 (PD-L1) on exosome surfaces to trigger or modulate immune activity [144,145]. Cancer cells have been demonstrated to leverage this attribute by producing exosomes presenting Wnt proteins or integrins with the intention of activating receptors on neighboring cells to ultimately shape the tumor microenvironment (TME) [146,147]. Proteins carried within the exosome can play more direct roles within the target cells once taken into the exosome, which can include proteins with direct roles in activating or inhibiting certain pathways, as well as copies of certain receptors and membrane channels that can amplify the response of the target cells to ambient molecules [148]. EVs are effective in facilitating unconventional protein secretion (UPS) of factors that would otherwise not enter the endoplasmic reticulum (ER)-to-Golgi (ER-Golgi) conventional secretion pathways or survive the transport process [149]. Exosomes are notably more secure and directed in UPS, among other EVs, allowing them to fulfill distribution of protein signaling where otherwise improbable.

#### 4.3.2. Transportation of Lipids

Like proteins, lipid cargo can be found as part of the membrane as well as within the exosomes. Generally, lipid cargo mainly lends themselves to supporting the membrane integrity, exosome interactions, and exosome uptake. The variable composition of these lipids not only defines the functional capacity of exosomes to securely transport cargo but have also been found to potentially follow certain patterns that may uniquely reflect the origin and disease state of parent cells of the exosome [150]. Functionally, membrane lipids may concurrently serve as signaling molecules or cofactors for enzymes within recipient cells, as in the case of phosphatidic acid and ceramide [151]. Lipids carried within the exosome can be steroid hormones or functional lipolytic enzymes. Interestingly, exosomal transport of lipids has been implicated in spread of lipid-related diseases, not only by the action of these lipids in triggering inflammatory responses but also in directly affecting lipid metabolism and storage, suggesting a domino-like mechanism for the spread of these diseases [152]. Certain lipids within exosomes can also modulate exosome sorting and output in targeted cells, further expanding the capacity for induction of a chain-reaction signaling among the cellular microenvironment [153].

#### 4.3.3. Transportation of Nucleic Acids

Of the nucleic acid cargo, miRNAs followed by ribosomal RNAs (rRNAs) and lncRNAs, make up the primary composition [112]. miRNAs, with the capacity to directly hinder the translation of certain mRNA sequences, allow for powerful unidirectional communication mechanisms for processes regulating cell survival, extracellular matrix (ECM) remodeling, apoptosis, metabolic reprogramming, cellular transformation, and immune response [142]. lncRNAs are highly versatile molecules, capable of functioning as molecular scaffolds, molecular sponges, and multi-level modulators of gene expression in cells targeted by the exosomes [154,155]. Both subtypes are recognized to not only play important individual roles in cell communication but also appear to potentially have a regulatory relationship affecting exosomal sorting of each RNA species. rRNAs have been typically found as fragments among exosome cargo [156]. Although their purpose is not as multifaced as their other RNA counterparts, rRNA fragments are thought to serve certain roles in modulating transcriptional activity of ribosomes and induction of stress response [157,158]. Unlike RNA, the importance of exosomal DNA transport remain poorly understood—only supported by identification of several DNA types and DNA-associated proteins within the exosomes [159]. Typically, DNA has been detected in larger EVs, as opposed to exosomes. However, it is theorized that exosomal DNA packaging can be triggered as a cellular safety mechanism to circumvent stress responses associated with the damaged DNA.

## 5. Engineering Therapeutic Exosomes

Although endogenous exosomes may lack the potency to overcome advanced disease—and are frequently subverted by the TME—they represent a highly changeable therapeutic platform. Their tightly regulated biogenesis offers multiple checkpoints for bioengineering, enabling the amplification of specific cargoes or the enhancement of overall production. By leveraging these distinct regulatory points, engineered exosomes can be transformed into versatile, pleiotropic modulators of the GBA–GBM axis.

### 5.1. Engineering Cargo Molecules

**Figure 4:**
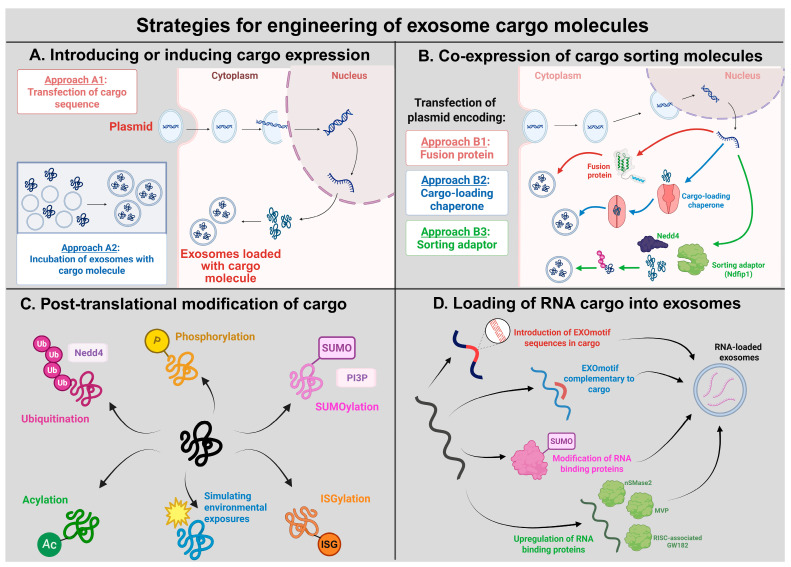
Strategies for engineering cargo molecules. The cargo intended for exosome transport can be modified in several ways that can enhance uptake, including (**A**) introducing via incubation or inducing expression of cargo molecules; (**B**) upregulating the co-expression of cargo sorting molecules, like fusion proteins, cargo-loading chaperons, and sorting adaptors for exosome sorting, alongside the cargo itself; (**C**) post-translational modification of the cargo molecules, such as ubiquitination, phosphorylation, SUMOylation, acylation, ISGylation, and specific environmental exposures; and (**D**) RNA uptake-specific strategies via introduction of EXOmotif sequences into cargo RNA or complementary to cargo RNA and modification or upregulation of RNA binding proteins for sorting. This figure was created using BioRender.

The typical strategy, which is employed to directly induce the expression of exosomes in a laboratory setting, is transfection of the exosome-producing cells [160]. This can be done through incubation with the molecules of interest, often coupled with a process that enhances their uptake into the cells. This exogenous loading process is more common and effective for introducing cargo molecules into a sample of exosomes that are produced and isolated earlier on [161,162]. This is to successfully introduce different biological molecules and is notable for the loading of synthetic drug compounds or nanomolecules that the cell would not naturally produce. A simple strategy to indirectly increase the integration of a certain cargo into exosomes is to increase the presence of that particular macromolecule in the intracellular environment (Figure 4).

For more selective loading of biomolecules and to ensure that neither cargo nor the exosomes are damaged by passive loading processes like electroporation, transfection of the cells with plasmids coding for the gene of interest is a more reliable approach. The transient increase in nucleic acids or peptides production through this process has been found to result in a higher proportion of exosomes produced containing the wanted molecules. The use of gene editing techniques to integrate copies of the gene sequences into the cell genome has been found to generate a more stable increase in gene expression. Introduction of particular promoter sequences in the cell can further enhance the expression of the genes. However, a key issue with these approaches is the risk of upsetting biological homeostasis or inducing cytotoxic effects with abnormally high rates of otherwise unrestricted expression of a particular product. The use of regulatory promoter sequences can help establish an inducible expression system that allows regulation of expression to specific instances or cellular contexts, especially in a physiological setting.

Beyond enhancing product availability, transfection can also be a viable approach in directly enhancing the packaging of the products. Encoding particular motifs into the code for the molecule of interest to produce fusion macromolecules can help enhance their transport to the site of exosome packaging and facilitate subsequent uptake. This strategy can be partnered with the expression or introduction of cargo-loading chaperone molecules that recognize particular biomolecule motifs [163]. One approach involved generating a fusion protein from MS2 bacteriophage coat protein and lysosomal-associated membrane protein 2b (Lamp2b, which is predominantly expressed in skeletal muscle and has a role in autophagy) or the vesicular stomatitis virus glycoprotein (VSVG, which is strongly implicated in several parts of exosome biogenesis), while the associated MS2 stem loop was engineered into cargo RNA 3′ untranslated regions (3′ UTRs). When applied, this Targeted and Modular EV Loading (TAMEL) approach resulted in as much as a six-fold enrichment of target RNA loading, based on particular proteins used in fusion [164,165]. Fusion of Lamp2b or tetraspanins (which belong to a family of proteins with four transmembrane domains and two extracellular loops) with proteins of interest is also a common strategy to aid localization to exosomes [166,167].

Compared to other major EV subtypes, exosomes seem to operate with the most specific cargo selection process. In this process, several post-transcriptional and post-translational modifications play a key role in guiding molecules for loading. Ubiquitination has been implicated in the sorting of proteins into exosomes, with some studies finding that about 13% of all exosomal proteins were ubiquitinated [168,169]. The ubiquitin marker can be recognized by the exosome biogenesis machinery and catalyze sequestration of the tagged molecule into ILVs. Attempting to increase ubiquitination via typical pathways or engineering ubiquitin tags onto proteins of interest has the potential to work but can yield non-specific results [170]. Several key exosome membrane proteins that are ubiquitinated rely on sorting adaptors for accurate transport to MVBs. In the case of Lamp2b, this involves association with Nedd4 (Neural precursor cell expressed developmentally down-regulated) family ubiquitin ligases, which bind to PPXY (proline, proline, any amino acid, and tyrosine) motifs to mark proteins for ubiquitination [171,172,173]. Proteins lacking the PPXY motif, like PTEN, benefit from the presence of Nedd4 family interacting protein 1 (Ndfip1) to bridge the interaction with the Nedd4 ubiquitin ligase [174,175]. Labeling of a target protein, Cre recombinase, with a WW (a small, about 35–40 amino acids, sequence module that binds proline-rich motifs) tag provokes its recognition by the L-domain-containing protein Ndfip, causing its ubiquitination and loading into the exosomes [176]. Upregulation of Ndfip1 has been found to correlate with increased presence of total protein and ubiquitinated proteins in exosomes, including those that were not previously found to be carried at the same level in exosomes [177]. Overexpression of other sorting adaptors like ALIX has been associated with similar increases in protein packaging [156].

Similar cargo modifications include the addition of SUMO, which is notable for its capacity for protein sorting, enhancing PI3P association with ESCRT machinery, and modulating the binding capacity of the exosomal miRNA export proteins such as heterogeneous nuclear ribonucleoprotein A2/B1 (hnRNPA2B1) and hnRNPA1 [178,179]. Inducing overexpression of UBC9 (ubiquitin-conjugating enzyme E2I), which facilitates transfer of the activated SUMO protein to target molecules, has been found to increase SUMOylation of hnRNPA1 and subsequent export of lncRNAs [180]. Phosphorylation is a versatile modification that can induce a host of effects, some of which may affect exosome cargo recruitment. While relatively higher proportions of phosphorylated proteins have been observed among exosomes than in the larger cell, the specific role of phosphorylation may be dependent on broader contexts, having been shown to hinder the recruitment of crystallin alphaB protein [181]. In some cases, localization to MVBs has been dependent on both phosphorylation and ubiquitination of the protein.

Carbohydrate modifications are less diverse among exosomal molecules but have been demonstrated to play important roles in molecular trafficking and cellular recognition. Oligomeric glycoproteins seem to depend on carbohydrate modifications for cargo sorting [182]. Preventing acylation of an oligomeric protein was found to have reduced their association with exosomal proteins involved with biogenesis [183]. Interferon stimulated gene 15 (ISG15) is a ubiquitin-like protein and conjugation of ISG15 to its targets is ISGylation, which has function in multiple cellular processes, including exosome secretion. Other modifications include palmitoylation, which enhances exosome release, and ISGylation, promotes lysosomal degradation of MVB proteins [184,185]. While a variety of cargo molecule modifications have been found to induce certain exosomal loading processes, many can have unintended or contradictory effects that occur based on certain cellular contexts. To exercise artificial control over the cellular contexts, a study found that the cloning of certain hypoxia-responsive elements induced gene expression patterns paralleling hypoxic response, thereby modifying both rates of expression and post-translational modification [186]. Based on this model, loading efficiencies can be improved by using relevant sensor elements to model other types of cellular responses, such as oxidative stress or pathogenic interaction, which have been shown to enhance miRNA loading.

As mentioned earlier in this section, strategies to enhance RNA loading either involve modification of the RNA molecules themselves to facilitate interaction with RBPs or to upregulate the activity of the RBP themselves. The former approach can be a natural result of certain motifs present in RNAs that have been found to function as cis-acting elements associated with loading in exosomes (therefore dubbed as EXOmotifs). Introduction of these known sub-sequences into target RNA can potentially facilitate targeting of these sequences [187,188]. Alternatively, generation of functional RNA aptamers (which are short single-stranded RNA molecules bind to specific targets, including proteins, small molecules, and even cells) that contain both the EXOmotifs and motifs that bind to target RNA sequences would help broaden the range of loadable-RNA sequences for therapeutic use. An interesting combination of these two approaches includes inserting the target RNA into the 3′ end of the pre-miRNA-451 backbone that binds directly to the exosomal RNA sorting protein Argonaute 2 (Ago2) [189]. Modification of the 3′ end of RNA sequences with certain tags has also been found to enhance uptake. Uridylation of the 3′ end of miRNA has been shown to be well associated with miRNA sorting [190].

The second group of strategies involves targeting the proteins involved with RNA sorting. Of these proteins, neural sphingomyelinase 2 (nSMase2) may be most well-known for its role in the process. Upregulation of nSMase2 was found to increase the number of exosomal miRNAs [191]. Cancer-related hyperactivity of other exosomal proteins such as major vault protein (MVP), RNA-induced silencing complex (RISC)-associated GW182 and Ago2, and myoferlin has also been linked to increased miRNA distribution to exosomes [192,193,194,195]. Post-translational modifications of these proteins are also known to have important effects on RNA sorting. O-GlcNAcylation and SUMOylation of hnRNPs have been found to correlate to their ability to recognize certain RNA motifs and facilitate their sorting [196,197,198]. Modification of RNA sorting proteins, when paired with RNA sequence modifications, can become a powerful strategy to enhance nucleic acid uptake. One group was able to concurrently (a) insert a C/D box, which is characterized by a bipartite pseudo-symmetric structure that contains box C (RUGAUGA) and D (CUGA), into the 3′ end of a target RNA transcript; (b) form a protein complex of C/D box-binding L7Ae and major exosomal packaging tetraspanin CD63; and (c) introduce cytoplasmic transfer assistant Connexin 43 to shuttle the bound miRNA sequences to the exosome for packaging [199,200,201]. Further bioinformatics analysis will be needed to identify more RBPs and correlating motifs that allow for expansive cargo selection processes.

### 5.2. Engineering Exosome Biogenesis

The primary pathway through which exosome biogenesis occurs is the ESCRT-dependent pathway, the core of which are ESCRT proteins 0, I, II, and III. Knockdown of certain ESCRT subunits has been demonstrated to decrease the size and rate of exosome release by cells [202]. However, there have not been well-defined strategies for directly enhancing the activity of ESCRT proteins. Overexpression of the ESCRT protein, while theoretically conducive to increased exosome production, was shown to result in inhibition of exosome production in certain cases [203,204,205].

Indirect modulation of the ESCRT-dependent exosome biogenesis pathway has been investigated through targeting of accessory proteins. The Ras/Raf/mitogen-activated protein kinase (MAP2K or MEK)/extracellular signal-regulated kinase (ERK) signaling pathway is a primary axis for regulating exosome production [206,207]. Conversely, inhibition of Ras farnesyltransferase enzymes has been observed to decrease exosome output [208,209]. Equally, mutant K-Ras in colorectal cancer cells have been tied to increased production of exosomes carrying certain miRNA products [210]. One mechanism through which this pathway takes effect is by upregulating the transcription of hnRNP H1, which corresponds to an increase in Hrs, ALIX, and Rab271 expression, while also simultaneously enhancing Ras signaling via facilitation of A-Raf translation [211]. The activity of the parallel signal transducer and activator of transcription 3 (STAT3) factor signaling pathway and upstream activator endothelin A receptor (ETA) has also corresponded to increases in exosome production, largely due to the increase in Rab27 activity, further strengthening the role of this axis of signaling in managing ESCRT-mediated exosome production [212,213].

The Rab proteins are a common target for indirect manipulation of ESCRT activity, due to the multifaceted role of this family in MVB transport. In cancers that use exosomes to facilitate their progression, Rab27a/b are commonly upregulated [214]. In multiple myeloma, this upregulation has been found to be a product of enhanced activity of the glutamate antiporter system, which also has a notable effect on tumor susceptibility gene 101 (TSG101) protein, ALIX, and vesicle-associated membrane protein 7 (VAMP7) [215]. Rab7 is also a key member of this family of proteins, the depletion of which in non-small-cell lung cancer (NSCLC) and breast cancer has affected MVB formation by disrupting the machinery for ER-endosome interplay [216]. The exact role of Rab7 and the corresponding fates of the MVBs it helps form are still under dispute, as some evidence suggests that it acts antithetically to Rab27 [217]. While predominant role of Rab7 is thought to ultimately involve fusion of MVBs with lysosomes, the intervention of other Rab proteins (like Rab31) or guidance of accessory proteins may alter this fate [218]. A Rab7 adaptor protein known as the Rab-interacting lysosomal protein (RILP) is crucial in mediating the transfer of Rab7-associated MVB to dynein motor proteins after MVB maturation and influencing determination of fate of the ILVs within. Cleavage of this RILP protein has been associated with increased packaging of miRNA and bias towards exosomal release of ILVs [218]. Modification of RILP certainly has potential as another site of ESCRT modulation.

Release of exosomes in the ESCRT-dependent pathway is the primary role of ESCRT-III. ALIX is widely recognized for its role in ESCRT-III recruitment. The activity of ALIX also seems to be tied to the regulatory mechanisms of the Ras/Raf/ERK pathway as well. The functional capacity of ALIX depends on the interaction of its Bro1 domain (with approximately 390 amino acid residues and boomerang shape for binding to ESCRT-III proteins for sorting function) and the charged multivesicular body protein 4 (CHMP4) to initiate membrane remodeling and link cargo molecules to ESCRT machinery. This cargo is transferred through ALIX from syntenin complexes, which binds to the variable (V) domain of ALIX with a YPXL motif [114]. The protein/Zonula occludens 1 (PDZ) domain of syntenin is a key regulatory site for the activity of syntetin and dictates the stability of the syntetin-ALIX complex [219]. PDZ is typically bound by various tetraspanins (TSPNs), each of which can induce certain effects enhancing or restricting exosome output. For example, TSPN6 is thought to interact with the syndecan (SDC)-bound syntenin to negatively regulate exosomal release, potentially by determining MVB fate [220,221]. Following the role of the ALIX-syntetin complex, disassembly of the ESCRT-III machinery and completion of ILV scission is carried out by vacuolar protein sorting 4 (VPS4) ATPase, which acts within the same pathways initiated by ETA, but downregulates exosome production at higher levels of expression [215,222,223].

ESCRT-independent pathways depend on caveolin, hnRNP, and SMases as key regulatory points. Caveolins are overexpressed in cancers that use exosomes to promote chemoresistance and their progression among the colony [224]. The same study also found that hnRNPs are also important to this process. There are several examples of post-translational modifications that regulate the activity of these two proteins. Phosphorylation of caveolin-1 (Cav1) is particularly relevant, as it exposes the Cav1 scaffolding domain (CSD) to allow binding of hnRNPA2B1, permitting the sorting of any associated RNA molecules [180]. SUMOylation and O-GlcNAcylation of hnRNPs enhances interaction with miRNAs [197]. Cav1 has also been known to be upregulated by action of wild type p53 [225]. The exosomal production resulting from upregulated Cav1 was found to be modified by acetylation of p53 [226].

SMases are common targets for inhibitors functioning on similar axes as ALIX and TSPNs, affecting processes such as budding of MVBs or exosome release by blocking the effect of the enzymes on membrane lipid composition [227,228]. The lipid composition of exosomes, in general, can have important implications in their ability to not only leave and enter a cell, but also survive in their journey. Lipid rafts are an abundant feature of membranes that have multi-level impact on exosome biogenesis. They can serve as direct and selective interfaces for several proteins and RNA/RBP-RNA complexes with the exosome [229,230]. The ceramide molecules produced by SMases coordinate the coalescing of lipid raft domains during the budding of ILVs, thereby helping dictate the specific properties of exosome membranes, which also serve as anchoring areas for some larger biomolecules.

Determination of exosome fate is a pivotal step for exosome release dynamics, given that much of the exosomal biogenesis and cargo sorting process can be highly similar between MVBs that approach lysosomal degradation or release as exosomes. The BLOC one-related complex (BORC)—ADP-ribosylation factor like member 8 (ARL8)—homotypic fusion and protein sorting (HOPS) or simply BORC-ARL8-HOPS pathway is a key axis in guiding MVBs for lysosomal degradation through facilitation of organelle transport and fusion with the help of effectors such as pleckstrin homology domain-containing family M member 1 (PLEKHM1) and HOPS [231]. Disruption of this pathway was found to prevent MVB-lysosome fusion, redirecting ILVs towards exosome secretion, and enhancing their release. MVB bias towards lysosomal pathway has also been associated with activity of mechanistic target of rapamycin complex 1 (mTORC1), the inactivation of which accelerated exosome release [232].

Fusion of MVB with the cell membrane is dependent on the structural support of several groups, such as SNAREs, cytoskeletal dynamics, and Ca^2+^ signaling pathways. SNARE proteins serve as gatekeepers that carefully regulate the fusion of vesicles with the plasma membrane. Modulating the expression of certain SNARE proteins and their constituent domains can result in a range of effects, unique to each SNARE, presenting a range of modulatory inlets to block or facilitate exosome release [233,234,235]. SNAREs are also modified by the activity of STAT3, which induces phosphorylation of pyruvate kinase M2 (PKM2), which in turn triggers phosphorylation of Q-SNARE protein called the synaptosomal associated protein of 23 kDa (SNAP23), thereby promoting exosome release [236]. SNARE proteins tightly coordinate with cytoskeletal components, for which well-characterized actin and myosin inhibitors can stimulate EV release [237]. Rho-associated kinases (ROCK) also serve as an important regulatory point for not only cytoskeleton activity, but also ERK activity, which influences exosome biogenesis at several axes [238].

Numerous cellular factors can have overarching roles in defining exosome biogenesis processes [239]. Hypoxia has been known to modulate not only the process of exosome biogenesis but also cargo sorting. It has been shown that hypoxic environments upregulate specific Rab27a and suppress Rab7 and lysosomal-associated membrane protein 1/2 (LAMP1/2), thereby enhancing exosome release [212]. Oxidation of DNA damage response protein ATM (Ataxia-Telangiectasia Mutated) in hypoxia has also been linked to autophagy-associated exosome release, although this may introduce certain cargo into exosomes related to cellular stress response, potentially resulting in some unintended effects [240]. Induction of hypoxia inducible factor 1 (HIF1) with dimethyloxalylglycine is proposed to induce exosome generation, while its suppression seemed to reduce the hypoxic effect on exosome production [241,242]. This paints HIF1 as a potential hub for hypoxic response, specifically now regarding exosome biogenesis. HIF1 activity in cancer cells was found to be stabilized by exosomal lncRNA released by the tumor-associated macrophages (TAMs) that were exposed to extracellular lactate [243]. Lactate, and the low pH it can cause, is known to modulate exosome cargo sorting and MVB fusion [244,245]. Inflammation, from viral infection, tissue damage, hyperhomocystenemia, or other causes, has also been shown to increase exosomal release. Activation of the NOD (Nucleotide-binding Oligomerization Domain)-Like Receptor Protein 3 (NLRP3) inflammasome inhibited association of the MVB with lysosome, thereby increasing the proportion of MVBs producing exosomes [246]. Ca^2+^ influx into intracellular space is commonly associated with cytoskeletal activities, including exosome release, due to its importance as a co-factor for several steps of the process involving proteins like ESCRTs, ALIX, and nSMase2 [247]. The Ca^2+^ sensory modules of the stromal interaction molecule 1 (STIM1) and the pore-forming protein Orai1 were found to be involved in suppressing exosome release at low levels from hepatocellular carcinoma (HCC) cells [248,249]. Viral infections like Hepatitis C have been found to not only enhance exosome production by inflammatory pathways, but also through stimulation of Ca^2+^-releasing mechanisms.

### 5.3. Enhancing Exosome Transport and Targeting

**Figure 5:**
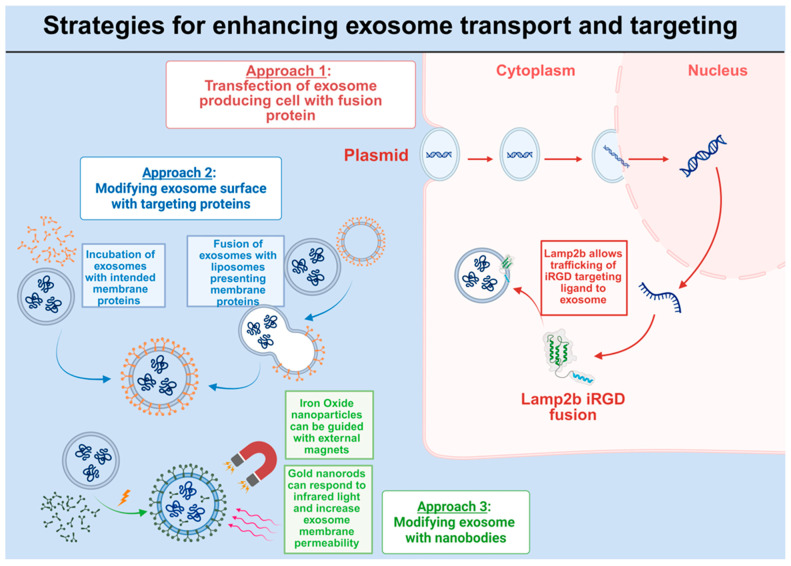
Strategies to enhance exosome transport and targeting. Exosomes can be modified in several ways that can enhance targeting based on specific cell or receptor types, including (1) inducing expression of fusion molecules that pair targeting molecules with exosome-associated proteins to encourage integration of the targeting molecules into newly formed exosomes; (2) modifying previously isolated exosomes either by incubation with targeting molecules or fusion with liposomes designed to present the targeting molecules; and (3) the integration of nanoparticles to facilitate external manipulation of exosome targeting via exposure to magnetism or radiation. This figure was created using BioRender.

The capacity of exosomes to deliver cargo to target tissues with precision defines their therapeutic potential. However, exosomes are naturally known to accumulate in non-target organs such as the liver, spleen, and kidneys [250]. Furthermore, the cargo within exosomes can produce negligible or negative effects if not received by the right cell population. The exosome membrane provides a malleable platform for engineering trafficking mechanisms that enhance the specificity and stability. Modification of surface proteins can be executed by genetic engineering of donor cells, fusion of targeting ligands, and direct biochemical modification of exosome surfaces (Figure 5).

A common approach involves modification of donor cells to produce fusions of targeting ligands and exosomal membrane proteins by transfecting exosome-producing cells with the modified gene. Lamp2b is a prime example of a membrane protein recruited to exosomes for its functional properties in biogenesis. Given its strong presence on the exosomal membrane, it becomes a prime target for fusion with targeting proteins such as rabies virus glycoprotein (RVG) that targets acetylcholine receptors in neural tissue, or the internalizing RGD (iRGD) peptide (a cyclic peptide of 9 amino acids with RGD sequence) that targets integrins on tumor cell membranes [251,252,253]. The Lamp2b iRGD fusion protein has naturally been found to be overexpressed in exosomes produced by immature dendritic cells and contributed to a successful delivery mechanism for doxorubicin to breast cancer cells [252]. This strategy can also help selectively expand targeting profile of exosomes in cases where fusion involves multiple single-chain variable fragment antibodies targeting different ligands [254]. While engineering of membrane proteins is a promising strategy, the integrated proteins are vulnerable to degradation during transport and can even be a detriment to the membrane integrity of the exosomes. Introducing certain modifications to the proteins can help prevent degradation [255]. Glycosylation of a fusion protein built from LAMP2b at several positions not only conferred a protective effect but also enhanced integration of the fusion protein into exosomal membranes [255].

More direct approaches of modification allow for introduction of new molecules into the existing exosome membrane. One of such strategies is modification through exosomal membrane fusion with external liposomes presenting the biomolecules of interest. Alternatively, exosomes presenting biomolecules of interest can be enhanced by fusion with phospholipid liposomes that improve the stability and circulation time of the modified exosomes [256]. Methods of producing fusion molecules from already existing manufactured proteins involve chemical reactions that result in fusion proteins. One example of this approach involves reacting dioleoylphosphatidylehtanolamine (DOPE) N-hydroxysuccinimide (NHS) with RVG to produce a fusion of the DOPE membrane protein with RVG [257]. When incubated with exosomes, the modified delivery vehicles induced a greater effect in neural tissue, indicative of successful RVG integration. These approaches are not only easier to implement but are also less restrictive than cell-based processes.

The introduction of synthetic nanoparticles into bioengineering technologies enhances opportunities for exosome modification. The precise customization capabilities of nanoparticles allow for selective targeting of exosomes. For example, nanobodies (smallest functional single-domain antibodies) can be designed to act as antibodies and embedded into the membrane of exosomes with the aid of anchoring peptides like glycosylphosphatidylinositol (GPI) [250]. Nanobody-modified exosomes have proven useful in delivering biomolecules and small molecule therapeutics to cell types of interest [258]. Conjugated surface proteins, similar in function to the fusion proteins described above, can also be generated using nanopartcies to similar efficacy without altering EV morphology or protein composition [259]. The properties of certain nanoparticles open new avenues for targeting. For instance, iron oxide nanoparticles can be integrated into exosomes using ultrasound or electroporation and subsequently be guided using external magnetic guidance [260,261]. Beyond targeting, gold nanorods integrated into cell membranes can respond to selectively delivered infrared light by heating up, thereby increasing the exosome membrane permeability during cellular intake [262].

Despite their promise, the process of exosome engineering does incur potential drawbacks. On one hand, while exosomes may possess tissue-specific targeting and biocompatibility qualities by virtue of their parent cells, they can also carry undesirable molecules that may contribute to unwanted effects in applications external to the tissue of origin. Conversely, modifications introduced to the exosomes, while capable of broadening exosome utility, require careful optimization to prevent imbalance of surface membranes and exosome constitution.

Broadly, despite strong preclinical promise, several translational barriers limit the near-term clinical deployment of exosome-based therapies. Manufacturing scalability remains a major challenge, as exosomes are produced in low yields and require complex, labor-intensive isolation and purification workflows that are difficult to scale up while preserving functional integrity. In parallel, substantial batch-to-batch heterogeneity arises from variability in cell source, culture conditions, and isolation methods, resulting in inconsistent cargo composition that complicates reproducible dosing and mechanistic interpretation. Finally, regulatory pathways for exosome therapeutics remain ill-defined, with exosomes occupying a gray zone between biologics and cell-based products; the absence of standardized criteria for characterization, potency, and release further hinders clinical translation and regulatory approval.

## 6. Exosome Engineering for Controlling GBA in GBM Preclinical Models and Patients

The intersection of EVs, GBA, and GBM represents a rapidly flourishing frontier in GBM research and therapy. As mentioned above, exosomes, being a specific subtype of EVs and nanovesicles (typically 30–150 nm), can mediate intercellular communication by transporting a complex cargo of proteins, lipids, and nucleic acids to distant recipient cells [263]. Their natural ability to cross the BBB and their low immunogenicity make them exceptional candidates for targeted GBM therapy [251,264,265]. GBA, the bidirectional communication system between the ENS and CNS, is significantly compromised by gut dysbiosis (an imbalance in the gut microbiota) in GBM patients and animal models [16,266]. Gut dysbiosis in GBM can create an immunosuppressive systemic environment that facilitates GBM progression and reduces the efficacy of standard treatments, TMZ, and immunotherapy [267]. The therapeutic strategy involves engineering exosomes to deliver payloads that specifically restore gut eubiosis, thereby reversing systemic immunosuppression and enhancing the anti-tumor immune response in the brain [268,269].

### 6.1. Exosome Biogenesis Gives the Blueprint for Their Therapeutic Engineering

Understanding both exosome biogenesis and key molecular machinery for cargo sorting, as described above in detail, is critical for developing a blueprint for their therapeutic engineering. Exosomes originate from the endosomal pathway, which provides a natural mechanism for packaging specific cargo. To reiterate, exosome biogenesis involves three main stages. First, endocytosis and early endosome formation begins with the inward budding of the plasma membrane to form early endosomes. Second, the membrane of the early endosome invaginates further, sequestering cytoplasmic contents and ILVs within the late endosomes, which are now termed MVBs. Finally, MVBs can fuse either with the lysosome for degradation, or they can fuse with the plasma membrane, releasing the ILVs into the extracellular space as mature exosomes [270]. The key molecular machinery for cargo sorting into ILVs/exosomes occurs via two main molecular mechanisms. ESCRT-dependent pathways play a major role in membrane budding, fission, and scission of the ILVs. ESCRT-independent pathways involving specific lipids, such as ceramide, or membrane proteins, such as tetraspanins (e.g., CD9, CD63, and CD81), which can also drive membrane curvature and cargo incorporation [105,271]. These elements are quite often exploited in specific exosome engineering strategies [272,273].

### 6.2. Exosome Engineering for Controlling Gut Dysbiosis in GBM

The inherent stability, low immunogenicity, and intrinsic targeting capacity of exosomes make them an ideal nanoplatform for therapeutic delivery across the GBA. Exosome engineering focuses on modifying the exosome’s surface (targeting) or its cargo (therapeutic payload). The most promising engineering focuses on enhancing exosome targeting specificity and therapeutic cargo loading [274]. For controlling gut dysbiosis in the context of GBM, the goal is to load factors that modulate the gut microbiome, reduce systemic inflammation, and enhance anti-tumor immunity in the brain [275].

#### 6.2.1. Cargo Loading Strategies

Exosomes can be loaded with payloads designed to restore eubiosis, such as microbiome-modulating small molecule drugs, anti-inflammatory miRNAs, or even SCFAs that are often deficient in dysbiosis [276,277]. Engineered exosomes are cell-derived nanovesicles with intrinsic advantages for drug delivery, including high biocompatibility, low immunogenicity, and the ability to cross physiological barriers. Recent research has leveraged these properties to develop precision oral delivery systems with gut-homing peptides that augment tissue-specific accumulation in the gastrointestinal tract (GIT). Common cargoes for GIT-targeted therapy include siRNA, miRNA, mRNA, proteins, and small molecules. Active loading enhances packaging efficiency and preserves therapeutic bioactivity. Exosome surface engineering is required for gut homing. Gut homing peptides are fused to exosome membrane proteins (e.g., Lamp2b, the tetraspanins CD9/CD63/CD81) to achieve tissue specificity. For gut targeting, integrin-binding and chemokine-responsive peptides have shown preferential adhesion to intestinal epithelial cells or Peyer’s patches. Surface modifications can be introduced via genetic fusion of ligands or chemical conjugation, allowing multivalent presentation for enhanced receptor-mediated uptake. Indeed, strategies for specific cargo loading into the exosomes can provide potential treatment of gut dysbiosis in GBM (Table 4).

#### 6.2.2. Challenges in Delivery of Exosomes Across the GBA

The delivery of exosomes across the GBA faces several challenges, including the difficulty in observing exosome movement in real-time, the need for a microfluidic-based system to mimic in vivo conditions, and the complexity of understanding the interaction between exosomes and the gut microbiome [283]. These challenges highlight the need for further research and development in this area to improve the delivery and functionality of exosomes in therapeutic applications [265]. While exosomes naturally cross the BBB to act on GBM [284], targeting the gut via systemic delivery (intravenous or IV injection) requires overcoming the challenge of non-specific accumulation in the liver and spleen. Therefore, engineered surface modifications for gut epithelial cell-specific uptake are crucial for an effective therapeutic approach to control gut dysbiosis.

### 6.3. Preclinical and Clinical Implications of Engineered Exosomes

Research on the direct use of engineered exosomes to treat GBM for correcting gut dysbiosis is still in its nascent stages, primarily focusing on animal models to establish the mechanism of the exosome-mediated GBA [285]. Recent research converges on the concept that gut dysbiosis plays a significant role in the initiation, progression, and therapeutic resistance of GBM. The emerging mechanism involves not only immune modulation but also exosome-mediated communication between gut-derived signals and the TME. Gut dysbiosis promotes GBM progression through a novel exosome-mediated axis involving metabolite signaling, immune system modulation, and direct tumor cell pathway activation. This reveals a targetable microbiome–tumor axis with significant potential for therapeutic intervention.

#### 6.3.1. Application of Engineered Exosomes to GBM Animal Models and Mechanisms

Studies using GBM orthotopic mouse models (e.g., murine GL261 or human xenografts) have established the link that tumor progression correlates with a shift in the gut microbial profile (e.g., increased *Bacteroidetes* and decreased *Firmicutes* or SCFA-producing bacteria) [285]. Gut dysbiosis manifests as increased pathogenic taxa and decreased protective taxa, contributing to a pro-tumorigenic immune environment. There is an exosomal communication loop manifesting that GBM-derived exosomes (GDEs) carry oncogenic signals (e.g., specific miRNAs or proteins like EGFRvIII) to promote an immunosuppressive TME [284]. Recent premises suggest that GDEs may also interact with gut immune cells or epithelial cells, acting as a “long-distance oncogenic signal” to induce systemic immunosuppression and gut dysbiosis, which in turn feeds back to promote tumor growth. Engineered exosomes loaded with anti-inflammatory cargo, such as the mesenchymal stromal/stem cell (MSC)-derived exosomes carrying miR-146a, have been evaluated in mouse models [286]. While generally focused on anti-neuroinflammation, this approach provides a template to assess that a therapeutically loaded exosome can modulate the systemic immune system, which is otherwise a key mechanism by which gut dysbiosis promotes GBM growth. Loading exosomes with potent anti-inflammatory miRNAs (e.g., miR-124a) or immunosuppressive drugs and targeting them to systemic immune cells or the gut can dampen the pro-tumor inflammatory loop fueled by GBA dysregulation [287]. This aims to reverse the immunosuppressive state and “re-educate” peripheral immune cells to mount an effective response against GBM.

#### 6.3.2. Future Clinical Translation of Engineered Exosomes to GBM Patients

While engineered exosomes show considerable promise as modulators of GBA in preclinical models of GBM, their clinical translation faces several practical challenges. Large-scale, Good Manufacturing Practice (GMP)-compliant production remains a major bottleneck, as exosomes are generated in low yields and require complex isolation and purification workflows that are difficult to scale up while maintaining consistent purity, potency, and functional activity [288]. Compounding this challenge is pronounced batch-to-batch heterogeneity, driven by variability in cell source, culture conditions, and loading strategies, all of which complicate reproducible dosing and mechanistic attribution. In parallel, achieving sufficient targeting specificity to disease-relevant gut or immune compartments remains critical to minimizing off-target effects [289]. The in vivo stability of engineered surface modifications and cargo further requires optimization, alongside rigorous characterization of pharmacokinetics, biodistribution, and long-term safety given the novel, multi-compartment mechanism of action across the GBA. Regulatory considerations add an additional layer of complexity, as exosomes occupy an ill-defined space between biologics and cell-based therapies, with no standardized criteria for characterization, potency assays, or release specifications. Ultimately, clinical translation will require standardized, scalable manufacturing pipelines, robust early-phase clinical trials to establish safety and dosing, and a strategic positioning of engineered exosomes as adjuvant therapies [273]. This can most plausibly be done in combination with immunotherapies such as immune checkpoint inhibitors, whose efficacy is closely linked to gut microbiome composition and systemic immune tone [290,291].

## 7. Engineered Exosomes as Multilevel Modulators of Gut Dysbiosis

Exosome-based interventions represent a sophisticated therapeutic paradigm that engages the gut ecosystem through a hierarchy of interdependent biological layers. Beyond simple cargo delivery, these engineered nanovesicles orchestrate a complex restoration of the gastrointestinal landscape by modulating localized immune responses, reinforcing the structural integrity of the epithelial barrier, and recalibrating the taxonomic architecture of the microbial community. To fully leverage their potential, research must transition from documenting phenotypic outcomes to dissecting the precise molecular interactions, which allow exosomes to restore coordinated homeostasis across the immune, mucosal, and microbial compartments. The therapeutic efficacy of these pleiotropic modulators of gut dysbiosis is inherently dictated by their biophysical properties, including surface-ligand orientation and the stoichiometric density of their encapsulated bioactive cargo (e.g., miRNAs, proteins, or small molecule metabolites). Consequently, the therapeutic potential of the engineered exosomes lies in their ability to serve as a programmable interface between host signaling pathways and the intraluminal environment. The following subsections synthesize contemporary preclinical evidence across diverse models of colitis and microbial dysbiosis to define the dominant mechanistic axes, ranging from the suppression of pro-inflammatory cytokine cascades to the metabolic priming of beneficial commensal species. In doing so, we highlight critical points of mechanistic convergence and provide causal evidence for exosomal efficacy in modulating gut dysbiosis, while critically addressing the current translational limitations in large-scale biomanufacturing and targeted delivery kinetics.

### 7.1. Immune Recalibration as a Systemic Mechanism of Benefits

Chronic intestinal inflammation is driven by dysregulated innate and adaptive immune responses, marked by excessive production of pro-inflammatory cytokines (e.g., tumor necrosis factor-alpha or TNF-α, IL-1β, IL-6, and interferon gamma or IFN-γ) alongside insufficient regulatory signaling mediated by IL-10 and transforming growth factor-beta or TGF-β. In the setting of gut dysbiosis, this imbalance promotes sustained mucosal injury and systemic immune skewing, which are processes implicated in cancer-associated immunosuppression and impaired antitumor immune surveillance. Accordingly, immune readouts such as cytokine profiles, macrophage activation states, and T cell polarization provide critical indicators of whether therapeutic interventions restore immune homeostasis rather than induce non-specific immunosuppression.

Across multiple experimental colitis models, exosome-based therapies consistently attenuate intestinal inflammation through immune reprogramming. MSC-derived exosomes and exosome-like nanoparticles reduce canonical inflammatory cytokines while increasing counter-regulatory mediators such as IL-10, with corresponding improvements in disease activity indices and colonic histopathology [292,293,294,295,296,297,298]. Macrophages emerge as a principal cellular mediator of these effects. In dextran sodium sulfate (DSS)-induced colitis models, exosome treatment is associated with reduced expression of M1-associated markers (e.g., inducible nitric oxide synthase or iNOS, CD86) and enrichment of alternatively activated macrophage markers (e.g., CD206, Arginase 1 or Arg1) in colonic tissue, as assessed by immunofluorescence, flow cytometry, or transcriptional profiling of isolated immune populations [292,293,296,298]. These phenotypic shifts are frequently accompanied by reduced macrophage infiltration or altered spatial distribution within inflamed mucosa, consistent with active modulation of macrophage state rather than passive dampening of inflammation. Supporting this interpretation, studies employing fluorescently labeled exosomes demonstrate preferential accumulation within intestinal and splenic immune compartments following systemic administration, paired with coordinated suppression of macrophage-derived inflammatory cytokines and induction of IL-10 at both transcript and protein levels [292]. Together, these findings indicate that exosomes act within relevant innate immune populations to reprogram inflammatory signaling in vivo.

Beyond innate immune modulation, several studies demonstrate that exosome-based therapies also influence adaptive immune responses, particularly regulatory T cell (Treg) abundance and T helper 1 cell (Th1)/Th17 polarization. Mechanistic support is strongest in studies combining quantitative immune profiling (typically by flow cytometry of mesenteric lymph nodes, spleen, or colonic lamina propria) with pathway-specific perturbation or functional inhibition. In one representative study, exosomes derived from distinct macrophage polarization states were directly compared in a DSS colitis model, revealing that M2b macrophage-derived exosomes conferred the most pronounced protection [299]. This effect was associated with increased splenic Treg frequency and elevated IL-4 levels, and mechanistically linked to enrichment of the chemokine (C-C motif) ligand 1 (CCL1) within exosomal cargo and engagement of a CCL1/chemokine (C-C motif) receptor 8 or CCR8 signaling axis, supported by biodistribution imaging demonstrating intestinal localization after systemic delivery.

Parallel findings have been reported for exosomes derived from granulocytic myeloid-derived suppressor cells, which reduce disease severity while shifting T-cell composition toward a regulatory phenotype, characterized by decreased Th1 responses and increased Treg representation in mesenteric lymph nodes. Pharmacologic inhibition of Arg1 partially abrogated these effects, implicating arginine metabolism as a functional component of exosome-mediated immunoregulation rather than a passive marker of myeloid activation. Consistent with this mechanism, complementary in vitro assays demonstrated decrease in CD4+ T cell proliferation and IFN-γ secretion following exosome exposure [300]. Adaptive immune modulation has also been observed with dendritic cell-derived exosomes conditioned with IL-10 in the 2,4,6-trinitrobenzene sulfonic acid (TNBS)-induced colitis model in rats. Intraperitoneal administration after disease induction reduced expression of pro-inflammatory cytokine transcripts, including IL-2, IFN-γ, and TNF-α, while increasing IL-10 expression and Treg enrichment within the colonic lamina propria [301].

Collectively, these studies support a model in which exosome-based interventions recalibrate immune networks in colitis by modulating macrophage-T cell crosstalk and restoring adaptive immune balance, rather than targeting individual inflammatory mediators in isolation. Future studies should incorporate immune cell-specific uptake tracing and further loss-of-function perturbations of defined exosomal cargo to establish causal links between immune reprogramming and disease outcomes. Standardized comparisons of exosome source, dosing, and delivery route, alongside microbiota-dependence testing, will be essential to distinguish conserved mechanisms from model-specific effects and strengthen translational relevance.

### 7.2. Restoration of Epithelial Barrier Integrity and Mucosal Repair

Intestinal epithelial barrier dysfunction is a central pathological feature of inflammatory bowel disease (IBD) and gut dysbiosis, as loss of tight-junction integrity and epithelial damage permit microbial translocation, perpetuate inflammation, and amplify systemic immune activation. Barrier-related readouts include tight junction protein expression, epithelial permeability, preservation of goblet cell (specialized epithelial cells for secretion of mucus), and mucosal architecture. These represent critical indicators of whether therapeutic interventions address upstream disease drivers rather than downstream inflammatory sequelae. Across experimental colitis models, exosome-based interventions consistently restore epithelial barrier integrity through coordinated improvements in structural and functional measures. Treatment is associated with preservation of tight junction proteins (e.g., Zonula Occludens-1 or ZO-1, occludin, and claudins), maintenance of colon length, reduced histologic injury, and decreased intestinal permeability [294,297,302,303]. Functional barrier integrity is most commonly assessed using fluorescein isothiocyanate dextran (FITC-d) leakage assay, providing a quantitative measure of paracellular permeability that complements histologic scoring and junctional protein localization [304].

One study demonstrated that DSS exposure increased serum FITC-d levels and promoted microbial invasion of colonic tissue, consistent with barrier disruption [304]. Subsequent exosome treatment reduced permeability and bacterial translocation while restoring antimicrobial peptide expression, including lysozyme, defensins, and Ang4, supporting recovery of coordinated epithelial defense programs rather than isolated junctional repair [304]. Similarly, another DSS colitis study demonstrated preservation of ZO-1 and occludin localization along the epithelial border accompanied by significant reductions in FITC-d leakage, linking ultrastructural junctional integrity with functional barrier restoration [302]. Together, these complementary readouts support interpretation of barrier recovery as a systems-level re-establishment of mucosal homeostasis. Mechanistic studies further establish epithelial barrier restoration as a causal driver of therapeutic benefit in exosome-treated colitis models. In one of the most rigorously defined systems, MSC-derived exosomes carrying tumor necrosis factor-stimulated gene 6 (TSG-6) were shown to be both necessary and sufficient for protection in DSS and TNBS colitis. Exosome treatment preserved tight junction architecture, goblet cell abundance, and mucosal permeability, while genetic depletion of TSG-6 from donor cells abolished these effects; barrier integrity was subsequently restored by recombinant TSG-6 supplementation, establishing a direct cargo–phenotype relationship [294].

Complementary studies implicate exosomal RNA cargo as an additional layer of epithelial regulation. Exosome-associated circRNAs promote epithelial repair through regulation of claudin-1 expression via CCCTC-binding factor (CTCF)- and methyltransferase-like 3 (METTL3)-dependent pathways, linking epigenetic regulation to junctional stability [305]. Similarly, miRNA-mediated effects on epithelial junctions have been demonstrated in both in vivo and in vitro systems. A study reported that exosomal miR-181a enhanced expression of junctional and inflammatory regulatory proteins, including claudin-1, ZO-1, and inhibitor of NF-κB or IκB, in DSS-treated mice and LPS-injured epithelial cells [297]. Inhibition of miR-181a attenuated these protective effects, directly tying barrier restoration to a defined exosomal cargo rather than to vesicle delivery alone.

Beyond preservation of existing barrier structures, several edible exosome-like nanoparticle systems promote epithelial proliferation and regeneration, as evidenced by increased expression of cell proliferation and differentiation markers such as PCNA (proliferating cell nuclear antigen), CDX2 (Caudal-type homeobox transcription factor 2), and IGF-1R (insulin-like growth factor 1 receptor) and suppression of epithelial stress pathways, including p53 signaling [306,307,308]. Collectively, these findings position epithelial barrier restoration and regeneration as central mechanistic endpoints through which exosome-based therapies re-establish mucosal homeostasis and normalize gut-immune interactions. Despite these strengths, most studies rely on acute colitis models and bulk epithelial readouts and lack lineage-specific tracing or long-term assessment of barrier durability, limiting resolution of cell-type specificity and persistence of barrier repair.

### 7.3. Direct and Indirect Microbiome Modulation

Alterations in gut microbial composition are a defining feature of dysbiosis, but compositional shifts alone are insufficient to establish causality. In the context of exosome-based interventions, microbiome readouts must therefore be interpreted with respect to whether microbial modulation reflects a primary, direct effect of exosome–microbe interactions or a secondary consequence of restored epithelial and immune homeostasis. The most compelling evidence for direct microbiome modulation comes from plant-derived exosome-like nanoparticles, which demonstrate cargo-dependent, cross-kingdom regulation of bacterial function. Ginger-derived nanoparticles are selectively internalized by *Lactobacillaceae* through lipid-mediated interactions, after which their RNA cargo targets bacterial transcripts to increase production of indole-3-carboxaldehyde [309]. This microbial metabolite activates aryl hydrocarbon receptor (AhR) signaling and induces IL-22-dependent mucosal protection in the host epithelium, establishing a continuous mechanistic link between nanoparticle uptake by defined taxa, altered microbial metabolism, and epithelial barrier reinforcement.

A parallel mechanism has been described for garlic-derived exosome-like nanoparticles, which promote the growth of *Bacteroides thetaiotaomicron* through sequence-specific miRNA-bacterial gene interactions. Fluorescent co-localization assays confirmed nanoparticle uptake, while anaerobic culture experiments demonstrated enhanced bacterial expansion following exposure. These effects were abolished by scrambled miRNA controls, indicating dependence on intact RNA cargo rather than non-specific vesicle or nutrient effects [310]. In contrast, microbiome changes reported following mammalian or MSC-derived exosome therapy are more plausibly secondary to improvements in host inflammation and epithelial integrity rather than the result of direct microbial targeting. In several DSS colitis studies, MSC-derived exosome treatment was associated with partial restoration of microbial diversity and normalization of disease-associated compositional shifts, including reduced expansion of *Proteobacteria* and rebalancing of *Firmicutes:Bacteroidota* ratios [297,298,311]. These changes occurred alongside reduced mucosal cytokine expression and improved barrier integrity, and microbiome profiling by fecal 16S rRNA sequencing at endpoint revealed community restructuring consistent with a less inflamed intestinal environment.

For example, a study reported that MSC-exosome treatment reduced overrepresentation of pro-inflammatory taxa while increasing commensal anaerobes associated with barrier-supportive metabolism, in parallel with restored tight junction architecture and suppressed inflammatory signaling [297]. Another study observed similar compositional normalization alongside epithelial repair and reduced permeability, but did not demonstrate exosome uptake by bacterial populations or modulation of microbial gene expression, limiting inference regarding direct host-microbe communication [298]. Across these studies, microbiome analyses were temporally downstream of barrier restoration and immune reprogramming, making it difficult to determine whether microbial changes contributed causally to therapeutic benefit or instead reflected improved ecological niches following mucosal healing. Importantly, few mammalian exosome studies incorporate causal perturbations such as antibiotic depletion, fecal microbiota transfer, or exosome-microbe interaction assays. Consequently, while observed microbial shifts are biologically consistent and reproducible, they are best interpreted as secondary consequences of epithelial and immune normalization rather than evidence of direct microbiome targeting by mammalian exosomes [297,298,311,312].

### 7.4. Microbial Metabolite Signaling as a Mechanistic Intermediary

Microbial metabolites provide a functional bridge between microbial composition and host physiology, directly shaping epithelial integrity, immune responses, and systemic metabolism. Accordingly, metabolomic readouts of particularly SCFAs, indole derivatives, and bile acids offer mechanistically informative endpoints that extend beyond descriptive taxonomic shifts. Several exosome-like nanoparticle systems have been shown to modulate microbial metabolic output, most prominently SCFA production, thereby linking microbiome restructuring to host benefit. In a diet-induced dysbiosis model, citrus-derived exosome-like nanoparticles restored microbial balance while significantly increasing colonic acetate, propionate, and butyrate levels [312]. These metabolic changes coincided with reduced intestinal inflammation and improved epithelial barrier markers, supporting a role for enhanced SCFA production in mucosal recovery rather than passive reflection of altered taxa abundance. A similar relationship was observed with kidney bean-derived nanoparticles, which increased acetate and propionate concentrations alongside improvements in systemic metabolic parameters and inflammatory indices, accompanied by enrichment of SCFA-producing bacterial communities [313]. Marine-derived systems reinforce this paradigm: sargassum-derived nanoparticles were associated with SCFA enrichment, expansion of SCFA-producing taxa, and suppression of inflammatory signaling pathways in colitis models [302]. These studies are strengthened by designs that pair microbial community profiling with targeted metabolomic analyses (most commonly gas chromatography-mass spectrometry-based SCFA quantification) allowing microbial functional output to be assessed directly rather than inferred from relative abundance alone [312,313]. Such approaches provide stronger mechanistic grounding for microbiome-mediated effects on inflammation control and barrier maintenance.

Indole-based microbial metabolites provide some of the most mechanistically resolved examples of exosome-like nanoparticle-microbiome-host crosstalk. Ginger-derived exosome-like nanoparticles were shown to enter defined commensal bacteria and induce production of indole-3-carboxaldehyde, which activated AhR signaling and IL-22-dependent mucosal protection, establishing a continuous pathway from nanoparticle uptake to microbial metabolism and host epithelial defense [309]. Related mechanisms have been described for garlic-derived nanoparticles, which increased indole-3-propionic acid levels alongside *Lactobacillus* expansion and suppression of pro-inflammatory cytokines [314], and for Portulaca-derived nanoparticles, which promoted AhR-dependent reprogramming of CD4+ T cells through microbial indole signaling, resulting in improved immune balance and reduced intestinal inflammation [315].

**Figure 6:**
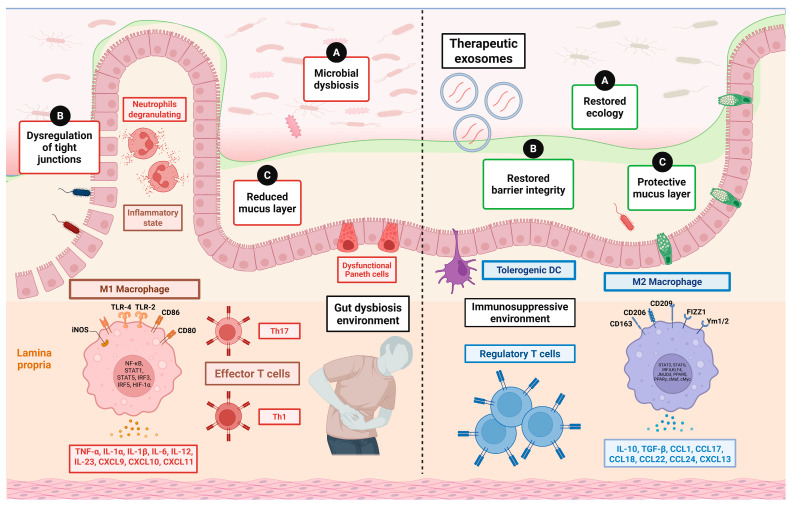
Exosome-based approaches for modulation gut dysbiosis. Both induction of gut dysbiosis (**left** side panel) and therapeutic exosome mediated amelioration of gut dysbiosis (**right** side panel) are depicted. Exosomes have been leveraged as therapeutic and delivery systems to restore gut eubiosis environment in several ways, including enhancement of commensal ecology, strengthened barrier integrity, restoration of mucus layers, M2 polarization of macrophage populations, upregulation of Treg populations, and enrichment of tolerogenic dendritic cell populations [316]. This figure was created using BioRender.

Metabolite-mediated host signaling extends beyond indole pathways. In mammalian systems, MSC-derived exosome treatment has been associated with altered bile acid composition and activation of farnesoid X receptor signaling, integrating microbial metabolic shifts with epithelial and immune regulation in colitis models [311]. Although the causal hierarchy in these systems is less fully resolved than in plant-derived nanoparticle studies, the findings support a broader framework in which exosome-based interventions influence host physiology through microbiome-derived signaling intermediates rather than direct antimicrobial activity. Despite strong associative evidence, many studies do not yet incorporate perturbative tests like metabolite receptor blockade, microbial depletion, or transfer experiments that would definitively establish metabolite causality. Future work integrating these approaches will be essential to distinguish primary metabolic drivers from secondary consequences of mucosal healing. Recent studies suggest invovelment of multiple molecular and cellular mechanisms in modulation of gut dysbiosis using exosome-based therapies (Figure 6).

### 7.5. Exosome Design Principles and Gut-Targeting Considerations

The therapeutic relevance of exosome-mediated gut modulation depends not only on biological activity but also on delivery efficiency, tissue targeting, and cargo stability. Accordingly, biodistribution, retention, and cargo validation represent essential translational readouts. Multiple studies provide direct evidence that exosomes and exosome-like nanoparticles preferentially accumulate within inflamed intestinal tissues, supporting injury-directed delivery rather than uniform systemic exposure. In DSS colitis models, systemically administered MSC-derived exosomes were fluorescently labeled and shown to localize predominantly to the colon and spleen-sites enriched for activated immune populations during intestinal inflammation. This localization coincided temporally with cytokine suppression and macrophage reprogramming, supporting the conclusion that exosomes access disease-relevant immune compartments rather than acting exclusively through circulating effects [292]. More refined localization analyses have been reported for macrophage-derived exosomes. In one study, exosomes derived from polarized macrophage subsets were fluorescently labeled prior to administration and directly visualized within inflamed colonic tissue using ex vivo imaging and histologic sectioning. This approach enabled correlation of vesicle localization with immune modulation and disease attenuation, strengthening claims of tissue-level targeting beyond inference from therapeutic outcome alone [299].

Edible exosome-like nanoparticles represent a distinct delivery paradigm in which gastrointestinal stability and mucosal retention are intrinsic properties of the system. Plant-derived nanoparticles have been shown to resist digestive degradation and persist within intestinal tissue following oral administration, as demonstrated by fluorescence-based tracking. In these systems, uptake by intestinal epithelial cells or commensal bacteria was directly visualized, providing spatial resolution that links oral delivery to local biological effects without reliance on systemic biodistribution [302,310,317]. Targeted engineering strategies further enhance specificity and therapeutic efficacy. Milk-derived exosomes functionalized with antibodies against mucosal addressin cell adhesion molecule-1 (MAdCAM-1)—an endothelial marker selectively upregulated in inflamed intestinal vasculature—exhibited increased colonic accumulation and superior attenuation of inflammation in a microplastics-induced colitis model compared with free cargo or untargeted carriers. The inclusion of comparator arms enabled attribution of efficacy to targeting-dependent delivery rather than nonspecific vesicle effects [318].

Beyond delivery, several studies have interrogated the molecular basis of exosome-mediated efficacy by testing the relative contribution of cargo classes. Across multiple systems, selective degradation of RNA cargo by RNase treatment markedly attenuated therapeutic benefit, whereas protease treatment had more modest effects, implicating RNA, particularly small non-coding RNAs, as dominant functional effectors [303,319]. These perturbation-based approaches provide mechanistic resolution even when individual miRNAs are not fully defined. Consistent with this framework, MSC-derived exosomes enriched in miR-181a improved epithelial barrier integrity and suppressed inflammatory cytokines in DSS-induced colitis, but these effects were significantly diminished by miR-181a inhibition in both in vivo and epithelial injury models, establishing miRNA-dependent efficacy [297]. Similarly, depletion of TSG-6 from MSC-derived exosomes abolished protection in DSS and TNBS models, while recombinant TSG-6 recapitulated barrier repair and immune modulation, demonstrating both necessity and sufficiency of this cargo component [294].

Despite growing evidence for therapeutic benefit, interpretation of exosome-based interventions remains constrained by heterogeneity in dosing regimens, cargo quantification, and biodistribution assessment. Variability in exosome source, loading efficiency, and delivery route complicates cross-study comparison and limits mechanistic generalization, underscoring the need for standardized experimental design and reporting frameworks [292,297,298,304,317]. Nonetheless, the increasing incorporation of cargo-perturbation and targeting strategies mitigates the “exosome black box” concern by anchoring biological effects to defined molecular payloads and delivery mechanisms, providing a clear-cut path toward translational development.

## 8. Evidence Supporting Frameworks on Therapeutic Exosomes for Modulation of GBA

Although an exosome–gut–GBM axis has not been established yet, converging preclinical evidence from non-oncologic CNS disease models supports the broader plausibility of vesicle-mediated gut–brain communication as a biologically meaningful pathway. Across multiple disease contexts, EVs and exosome-based interventions have been shown to influence neurologic outcomes in parallel with reproducible remodeling of gut microbial composition, intestinal immune tone, and microbial metabolism. While these findings do not demonstrate relevance to malignant CNS disease, they do provide a mechanistic framework for considering gut-targeted or gut-modulating exosome strategies as a hypothesis-driven avenue for GBM studies.

The most compelling causal evidence for exosome-mediated gut–brain crosstalk comes from a study employing mesenchymal stem cell-derived exosomes in intracerebral hemorrhage (ICH) models [320]. In a collagenase-induced ICH mouse model, systemic exosome administration significantly improved neurologic severity scores, reduced cerebral edema, and suppressed induction of apoptosis and inflammatory signaling centered on a TNF receptor-associated factor 6 (TRAF6)/NF-κB axis. Importantly, these CNS benefits were accompanied by partial restoration of gut microbial diversity, reshaping of taxonomic composition, and marked changes in fecal metabolomic profiles. Functional relevance of microbiome remodeling was directly tested through fecal microbiota transplantation (FMT): transfer of microbiota from exosome-treated donors conferred partial neuroprotection to antibiotic-depleted recipient mice, providing evidence that gut ecological changes actively contributed to neurologic recovery rather than reflecting a secondary correlate of CNS repair. Although exosome biodistribution to the brain was not directly quantified and some molecular details warrant further clarification, the inclusion of FMT represents a critical methodological advance beyond correlative microbiome analyses.

Broader mechanistic plausibility for vesicle-mediated gut–brain signaling is further supported by work in neurodegenerative disease, particularly AD, where interactions between gut dysbiosis, immune-derived exosomes, and neuroinflammation have been extensively characterized [321]. In this framework, exosomes originating from peripheral immune cells or the intestinal epithelium under dysbiotic conditions are proposed to traverse the blood–brain barrier and engage microglial Toll-like receptors (TLRs), activating mitogen-activated protein kinase (MAPK) and NF-κB signaling cascades that amplify neuroinflammatory damage [322]. Alterations in gut microbiota-derived metabolites, particularly SCFAs, were shown to influence exosomal cargo composition and membrane properties, thereby modulating vesicle stability and uptake by CNS cells [323,324]. Although much of this evidence remains preclinical and inferential, the AD literature provides a well-developed conceptual model linking gut dysbiosis, exosomal communication, and CNS immune activation, supporting biological plausibility of similar pathways in other neurologic contexts.

Evidence for bidirectional gut–brain coupling is also observed in PD models, where CNS-directed therapies are accompanied by reproducible normalization of intestinal immune and microbial states [325]. In a PD mouse model, intranasal administration of human umbilical cord-derived MSCs improved motor performance, preserved dopaminergic neurons, and attenuated glial activation while concurrently restoring microbial diversity and reducing expansion of pro-inflammatory taxa such as *Proteobacteria* and *Enterobacteriaceae*. These neurologic improvements coincided with enhanced gut barrier integrity, recovery of populations of goblet cells (that secrete mucus to protect and lubricate mucosal surfaces), and suppression of colonic NF-κB signaling. While exosomes were not isolated as the sole mediator and causality was not directly tested, the tight temporal association between CNS recovery and intestinal remodeling supports a bidirectional brain–gut axis in which neurologic injury and repair are linked to peripheral microbial states.

Additional studies further underscore the capacity of gut-derived vesicles to influence CNS biology, albeit with varying degrees of mechanistic resolution. In a colorectal cancer-associated postoperative cognitive dysfunction (POCD) model, exosome-like nanoparticles isolated from dysbiotic gut microbiota were internalized by hippocampal neurons and induced apoptosis, autophagy-linked ferroptotic stress, and cognitive impairment following systemic administration [326]. Although not directly relevant to malignant disease and focused on microbiota-derived EVs rather than therapeutic exosomes, this study provides a rare causal evidence that gut-derived vesicular cargo can engage neuronal death pathways and drive behavioral phenotypes. Similarly, nano-based delivery frameworks linking metabolic disease and PD emphasize the feasibility of exosome-mediated BBB crossing and the contribution of gut dysbiosis to neurodegenerative progression, though these analyses remain largely conceptual [327].

## 9. Engineered Exosomes for Enhancing Cell Death Mechanisms in GBM

In addition to ameliorating GBM through the gut–brain axis, systemic circulation of exosomes can also create opportunities for therapeutic impact at the tumor site itself. The poor prognosis and limited range of therapeutic options for GBM is in large part due to the formidable BBB, which severely restricts drug penetration, and the intrinsic resistance mechanisms of GSCs [328,329]. Exosomes, being the nano-sized (30–150 nm) extracellular vesicles released by virtually all cells, and their therapeutic engineering, have emerged as transformative delivery vehicles to overcome these barriers [291,330]. Their inherent ability to cross the BBB, low immunogenicity, and capacity to deliver diverse molecular cargo make them ideal candidates for enhancing the efficacy of therapeutics that induce regulated cell death (RCD) mechanisms in GBM cells [331,332]. The therapeutic strategy involves engineering these biological nano vehicles to efficiently deliver pro-death payloads (e.g., small molecule drugs, specific ncRNAs) directly to the tumor site, overwhelming the GBM cells to refrain from mounting anti-apoptotic and other anti-RCD mechanisms.

### 9.1. The Critical Role of Exosome Engineering for Targeted Delivery Therapeutics to GBM

The success of exosome-based GBM therapy hinges on engineering strategies that enhance both cargo loading and tumor-specific targeting [276]. While native exosomes can deliver their contents, maximizing the therapeutic effect requires engineering these nano vesicles to carry supra-physiological concentrations of death-inducing agents and ensuring they preferentially bind to GBM cells over healthy brain tissue [333].

One of the advantages of using exosomes as delivery vehicles is their targeted homing across the BBB. Exosomes can naturally cross the BBB, but active targeting is necessary to increase their accumulation at the GBM site [334]. Surface engineering is used to exploit GBM-specific molecular signatures [296]. There are two possible approaches. (i) In peptide/aptamer display, exosomes can be decorated with targeting moieties. For instance, fusion of the exosomal membrane protein Lamp2b with a c(RGD) peptide a cyclic version of the RGD motif with a sequence of Arg-Gly-Asp (which targets the αvβ3 integrin overexpressed on GBM vascular cells and GSCs) can significantly enhance targeted uptake and internalization [335]. (ii) In antibody-mediated targeting, exosomes have been engineered to display single-chain variable fragments (scFv) of antibodies (nanobodies) that specifically recognize receptors highly expressed on GBM cells, such as the epidermal growth factor receptor variant III (EGFRvIII), a common oncogenic driver in GBM [333,336,337]. This “lock-and-key” mechanism ensures the therapeutic cargo is delivered precisely to GBM, where it can initiate cell death.

### 9.2. Enhancing Cell Death Mechanisms in GBM via Engineered Exosomal Cargo

**Figure 7:**
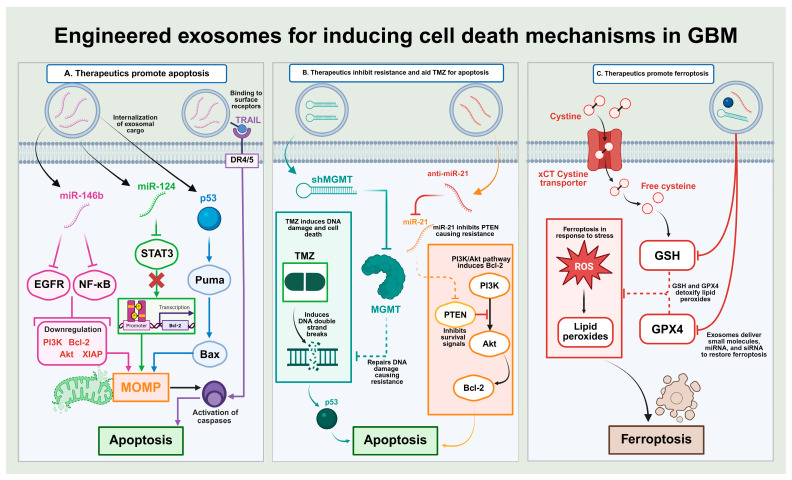
Exosome-based approaches for inducing RCD mechanisms (apoptosis and ferroptosis) in GBM. Exosomes have been leveraged as delivery systems in GBM models to induce apoptosis via several pathways, including (**A**) direct delivery of therapeutic cargo or regulatory surface proteins, including miRNAs that inhibit pro-tumorigenic pathways, regulatory proteins, or presentation of surface ligands that trigger anti-cancer pathways; (**B**) targeted inhibitory strategies to rout resistance mechanisms and re-sensitize tumors to other therapeutic approaches, involving shRNA and miRNAs that inhibit key resistance proteins; and (**C**) inducing a newer RCD mechanism such as ferroptosis. For induction of ferroptosis, the engineered exosomes deliver cargos that promote the intracellular lethal accumulation of iron-dependent lipid peroxides by silencing antioxidant defense systems such as glutathione (GSH) and glutathione peroxidase 4 (GPX4) pathway. This figure was created using BioRender.

Engineered exosomes serve as biocompatible delivery vehicles with high precision, designed to bypass the BBB and trigger robust cell death mechanisms in GBM. This therapeutic strategy operates through two main avenues: direct delivery of a cytotoxic agent and sensitization of GBM cells to existing therapies by targeting resistance pathways. In the first avenue, exosomes are precision-loaded with potent cytotoxic agents—ranging from pro-apoptotic proteins like TRAIL to specialized chemotherapeutics or miRNAs, which are delivered directly into the cytoplasm of malignant cells. By utilizing surface-engineered ligands that target GBM-specific overexpressed receptors (e.g., EGFRvIII), these vesicles ensure high local concentrations of the effector cargo. This targeted delivery minimizes systemic toxicity while maximizing the activation of caspase-dependent and independent death pathways within the tumor core. In the second avenue, exosomes focus on the strategic sensitization of GBM cells to conventional chemotherapy such as TMZ. Many GBM phenotypes evade programmed cell death by upregulating the DNA repair enzyme O6-methylguanine-DNA methyltransferase (MGMT), or by activating robust anti-apoptotic signaling loops like the PI3K/Akt pathway. Engineered exosomes can be programmed to carry inhibitory ncRNAs, such as siRNAs or miRNAs, that specifically silence these survival genes. By neutralizing the intrinsic resistance machinery in the tumor cells, these exosomal cargos restore the susceptibility of the tumor to existing therapies, effectively lowering the threshold required to trigger massive RCD mechanisms and prevent disease recurrence. Engineered exosomes can be used for inducing RCD mechanisms such as apoptosis and ferroptosis (Figure 7).

#### 9.2.1. Direct Induction of Apoptosis in GBM

Induction of apoptosis in GBM cells is characterized by specific morphological changes such as cell shrinkage, nuclear condensation, and DNA fragmentation, and biochemical changes mediated primarily by the activation of caspase proteases following therapeutic treatments [338,339]. Engineered exosomes can be used to deliver tumor-suppressive miRNAs that are often downregulated in GBM. For example, exosomal delivery of miR-146b has been shown in rat models to suppress GBM growth by silencing key oncogenic targets (e.g., EGFR and NF-κB) and subsequently activating the intrinsic pathway of apoptosis [340,341]. Similarly, engineered exosomal delivery of miR-124 can suppress cell proliferation and induce apoptotic cell death in GBM by targeting anti-apoptotic signaling pathways [342]. Another option is the use of engineered exosomes for pro-apoptotic protein delivery to GBM. For example, exosomes can be loaded with therapeutic proteins or peptides. One approach involves engineering exosomes to carry the full-length active form of the tumor suppressor protein p53 or pro-apoptotic proteins like TRAIL (tumor necrosis factor-related apoptosis-inducing ligand), overcoming the need for viral vectors and improving killing of GBM cells [343].

#### 9.2.2. Overcoming Chemotherapy Resistance in GBM

One of the major hurdles in GBM treatment is resistance to TMZ, often conferred by the DNA repair enzyme MGMT [344]. Engineered exosomes can be used to re-sensitize GBM cells to TMZ by delivering agents that inhibit MGMT to overcome this resistance mechanism. For example, engineered exosomes loaded with small interfering RNAs (siRNAs) or short hairpin RNAs (shRNAs) specifically targeting and knocking down the MGMT gene may demonstrate a remarkable ability to restore TMZ sensitivity in resistant GBM cell lines and xenograft models [345]. Further, engineered exosomes can be used for targeting anti-apoptotic pathways in GBM. GBM-derived exosomes naturally contribute to chemoresistance by transferring anti-apoptotic molecules (e.g., miR-21) to recipient cells [346,347]. Therapeutic exosome engineering can counteract this by delivering inhibitor molecules or competitive decoys, effectively shifting the cell fate balance toward apoptosis. The delivery of miR-21 inhibitors via targeted exosomes may enhance the efficacy of TMZ and RT [348].

#### 9.2.3. Induction of Novel RCD Mechanisms in GBM

Beyond the constraints of traditional apoptosis, which is frequently bypassed by both the GBM and GSC cells through the upregulation of anti-apoptotic proteins, recent research has pivoted toward the induction of alternative RCD mechanisms, most notably ferroptosis. Ferroptosis is an iron-dependent, non-apoptotic form of RCD characterized by the catastrophic accumulation of lipid peroxides and subsequent loss of plasma membrane integrity [349]. Engineered exosomes offer a sophisticated means of triggering this lethal pathway by delivering targeted molecular payloads designed to disrupt redox homeostasis in the tumor cells. These engineered exosomes can be loaded with small-molecule inhibitors or specific nucleic acids (siRNA or miRNA) that effectively deplete intracellular glutathione (GSH) or directly inhibit glutathione peroxidase 4 (GPX4), which is the primary enzyme responsible for neutralizing lipid peroxides [349,350,351]. By systematically dismantling these antioxidant defenses, engineered exosomes can drive the GBM cells toward irreversible oxidative damage and lipid-mediated lysis. This approach appears particularly promising for the eradication of GSCs, a subpopulation known to be notorious for therapeutic resistance and tumor recurrence. GSCs exhibit a unique metabolic vulnerability; they rely heavily on heightened antioxidant defense systems to survive the hypoxia and high-stress conditions of the TME. By targeting these specific metabolic dependencies through exosomal delivery, researchers can effectively overcome the intrinsic resistance of GSCs, offering a potent strategy to defeat both tumor progression and its post-surgical relapse.

### 9.3. Preclinical Validation and Clinical Outlooks

The preclinical evidence strongly supports the use of engineered exosomes as GBM therapeutics. MSC-derived exosomes (MSC-Exos) are a favored source due to their inherent tumor-homing ability and low immunogenicity [352,353]. In vivo studies using orthotopic GBM models can be designed to show that targeted, cargo-loaded MSC-Exos administered intravenously or locally can significantly suppress tumor growth, prolong survival, and reduce tumor cell proliferation compared to controls or free drug administration [354,355]. While clinical translation is still nascent, the focus is on safety, stability, and scaling up production of the engineered exosomes. As previously discussed, challenges remain in standardizing exosome isolation, ensuring large-scale GMP-compliant manufacturing, and precisely controlling the in vivo biodistribution [332,356]. However, the versatility of exosome engineering offers a powerful, customizable platform poised to revolutionize GBM treatment by directly inducing multiple, overwhelming RCD signals in these most aggressive cancer cells.

## 10. Conclusions and Future Directions

New strategies continue to emerge to counteract the factors that drive GBM progression, particularly those that enable tumor cells to evade RCD pathways. Recent advances in biotechnology, particularly in imaging as well as in exosome engineering to induce RCD in GBM and other tumors, are providing novel insights that may also be applicable to mitigating gut dysbiosis-mediated therapy resistance [357,358,359]. The gut microbiome exerts a critical influence on GBA and has significant therapeutic implications for many CNS disorders, including GBM [360]. By modulating systemic inflammation and immune responses, the gut microbiome can either exacerbate or attenuate GBM progression. Engineered exosome-based delivery platforms designed to correct gut dysbiosis represent a conceptually sound therapeutic approach, with the potential to restore microbial equilibrium, suppress inflammation, and enhance GBM sensitivity to apoptosis and ferroptosis. Owing to their intrinsic ability to transport bioactive cargos, exosomes present a versatile platform for modulating immune signaling and targeting disease specific pathways [361,362,363,364]. Nevertheless, challenges including low drug loading efficiency and limited targeting specificity must be addressed before exosome-based therapies can be fully optimized for clinical applications [365,366,367].

Gut dysbiosis promotes a systemic inflammatory milieu that can influence the GBM and its TME, thereby facilitating tumor growth. Based on recent findings, we have proposed that GBM progression may itself be driven partly by endogenous exosomes, and that this trajectory can be reversed by engineering therapeutic exosomes capable of resensitizing both the GBM and GSC cells to RCD, particularly apoptosis and ferroptosis. Accordingly, this article presents a timely and comprehensive synthesis of a novel conceptual framework juxtaposing two interconnected components of GBM pathogenesis: (i) gut dysbiosis acting through the GBA and (ii) the suppression of key RCD mechanisms. A major strength of this work is presenting an array of evidence to strengthen the rationale for a unified dual targeting strategy, in which engineered exosomes can simultaneously restore gut eubiosis and trigger apoptosis or ferroptosis within GBM tumor. To this end, we have provided an in-depth examination of exosome biology including biogenesis, cargo selection, and engineering approaches, thereby highlighting a solid landscape of therapeutic exosomes and EVs at large for GBM treatment. Additionally, we integrate discussions of GBA physiology, immune modulation, GSC resistance, and ever emerging exosome-based technologies, collectively addressing several of the most pressing challenges in GBM research.

Despite the therapeutic promise of exosome engineering for correcting gut dysbiosis and re-establishing susceptibility of GBM and GSC cells to apoptosis and ferroptosis, significant translational barriers remain. To overcome current limitations, future studies must focus on inconsistent and often low drug loading capacity, vesicle heterogeneity, rapid systemic clearance, suboptimal tumor tropism, and difficulties in large scale production, standardization, and storage. These characteristic challenges impair potency, reproducibility, and in vivo targeting specificity [368]. With that in mind, it is important to acknowledge that several of these challenges arise from the intrinsic pharmacokinetic behavior of exosomes in vivo, rather than solely from manufacturing limitations. Following systemic administration, exosomes are often rapidly removed from circulation within minutes, with reported half-lives as low as a few minutes, primarily through uptake by macrophages of the mononuclear phagocyte system, particularly hepatic and splenic macrophages [369,370]. Consistently, intravenously delivered vesicles preferentially accumulate in the liver and spleen, with secondary localization to the lungs, thereby limiting effective exposure of intracranial tumor tissue [161,371,372]. This clearance is mediated by recognition of surface phosphatidylserine, glycans, and integrin profiles on exosomal membranes by scavenger receptors and lectin receptors on macrophages, indicating that biodistribution is governed by active biological recognition rather than passive nanoparticle diffusion [373]. Consequently, poor tumor tropism and limited brain delivery represent pharmacokinetic constraints inherent to native exosomes. Ongoing advances in exosome bioengineering, including ligand display on vesicle membranes, hybrid exosome–liposome platforms, and microfluidic cargo loading technologies, aim to overcome some of these biological barriers by prolonging their circulation time and redirecting tissue targeting [373]. However, robust and clinically validated solutions are still required before these approaches can be fully implemented in clinical settings [374,375].

In summary, alterations in the gut microbiome influence GBM pathobiology through interconnected systemic and intratumoral immune pathways. Exosome engineering aimed at restoring gut eubiosis and activating RCD pathways in GBM offers a rational and potentially synergistic therapeutic trajectory that should encourage the scientific community to consider and explore further through rigorous preclinical and clinical studies. However, the clinical application of this platform hinges on the successful resolution of critical translational hurdles. The realization of the full potential of this dual-action therapeutic approach will depend on overcoming the key challenges in delivery, manufacturing, and standardization to ensure reliable translation of the engineered exosomes into effective treatments for gut dysbiosis and GBM.

## Figures and Tables

**Table 1:** Different diseases and disorders due to gut dysbiosis with increased and decreased bacteria.

Disease or Disorder	Increased Bacteria	Decreased Bacteria	Reference
Alzheimer’s disease (AD)	*Escherichia* and *Enterococcus*	*Lactobacillus*, *Bifidobacterium*, and *Ruminococcus*	[51]
Type 1 diabetes	*Bacteroidetes*, *Clostridium*	*Bifidobacteria*, *Lactobacillus*, *Prevotella*	[52,53]
Type 2 diabetes	*Bacteroidetes*, *Lactobacillus*	*Clostridium*, *Firmicutes*	[52]
Rheumatoid arthritis (RA)	*Verrucomicrobia*, *Lactobacillus*, *Streptococcus*, *Akkermansia* and *Proteobacteria*	*Bacteroidetes*, *Bacteroides*, and *Faecalibacterium*	[54,55]
Asthma	*Escherichia coli*, *Helicobacter pylori*, *Streptococcus*, and *Staphylococcus*	*Bifidobacterium* and *Lactobacillus*	[56]
Crohn’s disease	*Escherichia coli* and *Enterococcus* spp.	*Bifidobacteria* and *Bacillus lactic acid*	[57]
Nonalcoholic fatty liver disease	*Enterobacteriaceae* and *Enterococcus*	*Bifidobacterium* and *Lactobacillus*	[58]
Obesity	*Firmicutes*	*Fusobacteria*, *Proteobacteria*, and *Bacteroidetes*	[59]
Hypertension	*Firmicutes* and *Bacteroidetes*	-	[60]
Hepatitis and liver cirrhosis	*Enterobacteriaceae*, *Enterococcus*, *Staphylococcus aureus*, and *Saccharomyces*	*Lactobacillus*, *Bacteroides*, *Bifidobacterium*, and *Clostridium*	[61]

**Table 2:** Studies determining an association between gut microbiome and GBM via GBA.

Subject for Gut Microbiome Study	Association	Reference
GL261 mouse model	Increased *Firmicutes/Bacteroides* (F/B) ratio, *Verrucomicrobia phylum*, and *Akkermansia* genus	[75]
GL261 mouse model	Decreased SCFAs and neurotransmitters	[77]
GL261 mouse model	Decreased *Prevotellaceae*, *Rikenellacaea*, and *Helicobacteraceae*; increased *Burkholderiales*	[79]
Humanized mouse lines implanted with GBM	Increased *Bacteroides cellulosilyticus*	[81]
Human genome	Increased family *Peptostreptococcaceae* and genus *Eubacterium brachy* group	[80]
Humans	Increased *Fusobacterium* and *Akkermansia* Lacked SCFA-producing probiotics	[82]
Humans	Increased GBM acceleration after COVID-19 infection	[83]

**Table 3:** Biological utilities of exosomes and diversity of their cargos.

Location	Exosome Source	Exosome Function	Exosome Cargo	Pertinent Modifications	Reference
Immune system	Antigen presenting cells	Induce specific immune responses	MHC class I and II molecules	Expression of MHC molecules on exosome surface	[133]
Immune system	Mast cells	Promote DC maturation	Heat shock protein 60 kDa (HSP60) and HSC70	Enrichment of HSPs	[134]
Immune system	Bacteria-infected macrophages	Promote inflammatory response	Microbial antigens, and pathogen-associated molecular patterns	TLR-dependent activation	[135]
Nervous system	Neurons	Regulate synaptic activity	Proteins, nucleic acids, and lipids	Secretion regulated by Ca^2+^ influx and glutamatergic activity	[136]
Cardiovascular system	Cardiomyocytes	Regulate gene expression and cell signaling	mRNAs (1520 identified)	Modification of mRNA content under stress conditions	[137]
Cardiovascular system	Various cells	Promote angiogenesis and coagulation, modulate inflammation, and regulate vascular tone	Proteins, mRNAs, and miRNAs	Changes in concentration under stress conditions	[138]
Cancer	Tumor cells	Promote tumor progression, metastasis, and immune evasion	Oncogenes, pro-tumoral factors	Increased production compared to healthy cells	[139]
Stem cell maintenance	Various cells	Regulate stem cell function	Transcription factors and growth factors	Not specified	[140]
Tissue repair	Various cells	Facilitate tissue regeneration	Growth factors and signaling molecules	Not specified	[141]
Blood coagulation	Platelets, endothelial cells	Regulate coagulation processes	Coagulation factors and tissue factors	Not specified	[142]
Extracellular matrix remodeling	Various cells	Modify extracellular environment	Matrix metalloproteinases and other enzymes	Not specified	[143]

**Table 4:** Exosome cargo loading strategies for treating gut dysbiosis in GBM.

Cargo Loading	Mechanism to Make It Functional	Treating Gut Dysbiosis in GBM	Reference
Passive loading	Incubation, sonication, electroporation, and freeze–thaw cycles of purified exosomes with the cargo	Loading anti-inflammatory small molecules (e.g., specific antibiotics or phytochemicals) or SCFAs (e.g., butyrate) for delivery to the gut-associated lymphoid tissue (GALT)	[278,279]
Active loading via genetic engineering	Genetically modifying the parental cell (e.g., mesenchymal stromal/stem cells or MSCs) to overexpress a target cargo fused to an exosomal sorting tag (e.g., Lamp2b or CD63)	Loading specific miRNAs (e.g., miR-146a or miR-124, which have anti-inflammatory and anti-GBM effects) or lncRNAs that promote a healthy gut barrier function and shift the immune response from pro-tumor (M2-like) to anti-tumor (M1-like)	[280,281]
Surface modification for targeting	Functionalizing the exosome surface with specific peptides or aptamers to enhance targeting	Using gut-homing peptides or antibodies against specific receptors on gut immune cells (e.g., Peyer’s patches M cells or mucosal dendritic cells) to ensure accumulation at the site of gut dysbiosis	[282]

## Data Availability

No new data were created or analyzed in this study. Data sharing is not applicable to this article.
